# Comparison of the 12-month impact of COVID-19 and SARS on physiological capacity and health-related quality of life

**DOI:** 10.1186/s12890-023-02750-8

**Published:** 2023-11-14

**Authors:** Ken Ka Pang Chan, Susanna So Shan Ng, Grace Chung Yan Lui, Ho Sang Leung, Ka Tak Wong, Winnie Chiu Wing Chu, Tat On Chan, Karen Yee Shan Yiu, Eugene Yuk Keung Tso, Kin Wang To, Jenny Chun Li Ngai, Tommy Wing Ho Yip, Rachel Lai Ping Lo, Joyce Ka Ching Ng, Fanny Wai San Ko, David Shu Cheong Hui

**Affiliations:** 1 Division of Respiratory Medicine, Department of Medicine & Therapeutics, Prince of Wales Hospital, The Chinese University of Hong Kong, Hong Kong, China; 2 Division of Infectious Diseases, Department of Medicine & Therapeutics, Prince of Wales Hospital, The Chinese University of Hong Kong, Hong Kong, China; 3 Department of Imaging and Interventional Radiology, Prince of Wales Hospital, The Chinese University of Hong Kong, Hong Kong, China; 4 The Jockey Club School of Public Health and Primary Care, The Chinese University of Hong Kong, Hong Kong, China; 5 Department of Medicine, United Christian Hospital, Kwun Tong, Hong Kong

**Keywords:** 6-minute walking distance, Coronavirus disease 2019, Health-related quality of life, Lung function, Severe acute respiratory syndrome, High-resolution computed tomography

## Abstract

**Background:**

Little is known about the differences in medium to long-term recovery on spirometry, 6-minute walking distance (6MWD) and health-related quality of life (HRQoL) between COVID-19 and SARS.

**Methods:**

We performed a 12-month prospective study on COVID-19 survivors. The changes in dynamic lung volumes at spirometry (%predicted FEV_1_, %predicted FVC), 6MWD and HRQoL at 1–3, 6 to 12 months were compared against a historical cohort of SARS survivors using the same study protocol. The residual radiological changes in HRCT in COVID-19 survivors were correlated with their functional capacity.

**Results:**

108 COVID-19 survivors of various disease severity (asymptomatic 2.9%, mild 33.3%, moderate 47.2%, severe 8.3%, critical 8.3%) were recruited. When compared with 97 SARS survivors, 108 COVID-19 survivors were older (48.1 ± 16.4 vs. 36.1 ± 9.5 years, *p* < 0.001) and required less additional support during hospitalization; with lower dynamic lung volumes, shorter 6MWD and better physical component score. Both groups of survivors had comparable changes in these parameters at subsequent follow-ups. Both COVID-19 and SARS survivors had similar mental component score (MCS) at 6 and 12 months. COVID-19 survivors initially experienced less (between-group difference, -3.1, 95% confidence interval [CI] -5.5 to -0.7, *p* = 0.012) and then more improvement (between-group difference 2.9, 95%, CI 0.8 to 5.1, *p* = 0.007) than SARS survivors in the MCS at 1–3 to 6 months and 6 to 12 months respectively. Forty (44.0%) out of 91 COVID-19 survivors had residual abnormalities on HRCT at 12 months, with a negative correlation between the severity scores of parenchymal changes and 6MWD (r=-0.239, *p* < 0.05).

**Conclusions:**

COVID-19 survivors demonstrated a similar recovery speed in dynamic lung volumes and exercise capacity, but different paces of psychological recovery as SARS survivors in the convalescent phase. The severity of parenchymal changes in HRCT is negatively correlated with the 6MWD of COVID-19 survivors.

**Trial registration:**

This prospective study was registered at ClinicalTrials.gov on 2 November 2020 (Identifier: NCT04611243).

**Supplementary Information:**

The online version contains supplementary material available at 10.1186/s12890-023-02750-8.

## Introduction

The outbreak of coronavirus disease 2019 (COVID-19) emerged since the end of 2019 has resulted in over 769 million confirmed cases and 6.9 million deaths globally [1]. In Hong Kong (HK), there were 2,876,106 confirmed COVID-19 cases and 13,333 related deaths as of 12 April 2023 [2]. Although the extent of impairment, in terms of lung function, exercise tolerance, health-related quality of life (HRQoL) and radiological findings after recovery has been reported, the results were variable owing to differences in the admission criteria, treatment options and background demographics [3–11]. In HK, all patients diagnosed with COVID-19 before February 2022 were hospitalized for isolation purposes, irrespective of disease severity. This provided an opportunity for prospective longitudinal assessments of a full spectrum of asymptomatic to critical COVID-19 subjects.

In contrast, severe acute respiratory syndrome (SARS), caused by SARS-CoV-1, from late 2002 to mid-2003, resulted in 8,098 patients infected worldwide and 774 deaths [12]. Significant impairment in lung function, exercise capacity and health status among SARS survivors one year after illness were remarkably lower than in a normal population [13]. Although the case-fatality rate of COVID-19 was lower than that of SARS (0.4% vs. 9.6%), [14] no study compared the long-term recovery between COVID-19 and SARS survivors. A comparative study would characterize and inform long-term management strategies for emerging coronavirus infection. We hypothesize that the recovery trajectory of COVID-19 survivors followed a better trend than SARS survivors, taking into consideration varying disease severity and level of care at the baseline. Our study aimed to evaluate the differences in the medium to long-term longitudinal changes in dynamic lung volumes, exercise tolerance and HRQoL between COVID-19 and SARS survivors.

## Study design and methods

### Subjects

This is an ongoing longitudinal, follow-up study of patients with COVID-19 discharged after surviving the major outbreak in HK from 5 to 2020 to 17 September 2020 from three tertiary care hospitals. Consecutive patients, irrespective of their disease severity, consenting to this study were recruited. All recruited patients had laboratory confirmation of SARS-CoV-2 by reverse transcription-polymerase chain reaction of the respiratory specimens and were hospitalized for isolation purposes. Patients who refused to join the study or cognitively impaired were excluded. A historical cohort containing 97 consecutive SARS patients after surviving the major outbreak in 2003, who were discharged between 28 and 2003 and 26 Jul 2003, was included for comparison [13]. The sample size of COVID-19 survivors was not predefined, as there was no prior data comparing SARS and COVID-19 survivors available. Therefore, we prospectively recruited patients who were admitted during the first three waves of COVID-19 community spread in Hong Kong (between February and August 2020). The recruitment was stopped due to a low level of community spread between the third and fourth waves. A matching criteria between the two groups of survivors were not applied as the disease behaviour of COVID-19 was not completely revealed during the recruitment period.

This prospective study was approved by the Clinical Research Ethics Committees of the Chinese University of Hong Kong (CREC-2020.229) and registered at ClinicalTrials.gov (Identifier: NCT04611243). The study was performed in accordance with Declaration of Helsinki.

### Assessment

Following hospital discharge, COVID-19 survivors were evaluated at 1 to 3 months (a wide range of timings was allowed depending on the patient’s recovery and policy of social restriction), 6 and 12 months after discharge. During the visit, subjects were evaluated for baseline demographics, premorbid conditions, pulmonary function tests (measuring static and dynamic lung volumes, and diffusion capacity for carbon monoxide [DLCO]), 6-minute walk test (6MWT), and Medical Outcomes Study 36-Item Short-Form General Health Survey (SF-36) questionnaire. Respiratory comorbidities included underlying chronic airway or structural parenchymal diseases, including chronic obstructive pulmonary disease (COPD) and asthma (as defined by Global Initiative for Chronic Obstructive Lung Disease and Global Initiative for Asthma). This assessment package was performed according to international standards and used for assessing SARS survivors in 2003, [13, 15–20]with details explained in Appendix S1. The physiological and functional parameters assessed were compared to the normative data of HK [21–24]. Only patients who had completed both 6 and 12-month clinical follow-ups were included for statistical analysis.

#### High-resolution computed tomography (HRCT)

All COVID-19 survivors underwent a thin-section HRCT from the lung apices to the diaphragm at 12 months. The involvement of parenchymal changes was defined by a combination of consolidation, ground-glass opacity (GGO) and fibrosis, with the extent of involvement in each lobe graded, similar to previously described studies for SARS [25, 26]and COVID-19 [27, 28]. The radiological descriptive terms were based on the glossary defined by the Fleischner Society [29]. The details of the scanning protocol are explained in Appendix S2.

### Study outcomes

The primary outcomes were the between-group differences in the longitudinal changes of dynamic lung volumes, 6-minute walking distance (6MWD) and SF-36 scores from 6 to 12 months between COVID-19 and SARS survivors. The secondary outcomes included the within-group and between-group comparison of these parameters between COVID-19 survivors, SARS survivors and HK normative data at different time points, identification of factors determining the longitudinal changes, and the presence of residual radiological abnormalities on HRCT at 12 months and its correlation with baseline characteristics and various functional parameters.

### Statistical analysis

STROBE guideline was applied. Data were presented as n (%) or mean with standard deviation (SD), as appropriate. Independent *t*-tests and paired *t*-tests were used to compare the between-group and within-group differences in the changes in dynamic lung volumes, 6MWD and HRQoL over different time points respectively. Multivariate analysis (analysis of variance with repeated measures) incorporating the need for oxygen and corticosteroids during hospitalization, age and sex [17, 24] was performed to evaluate the potential determinants of these longitudinal changes. The need for additional support during hospitalization was included as the independent variable in the analysis as there was no shared severity score for both diseases. Spearman correlations were used to analyze associations among radiological changes, lung function tests and 6MWD at 12 months. Data were analyzed using SPSS version 26.0 (IBM Corp., USA). All statistical tests were two-tailed. Statistical significance was taken as *p* < 0.05. The details of the statistical analysis are explained in Appendix S3.

## Results

### Baseline characteristics of COVID-19 survivors

**Fig. 1:**
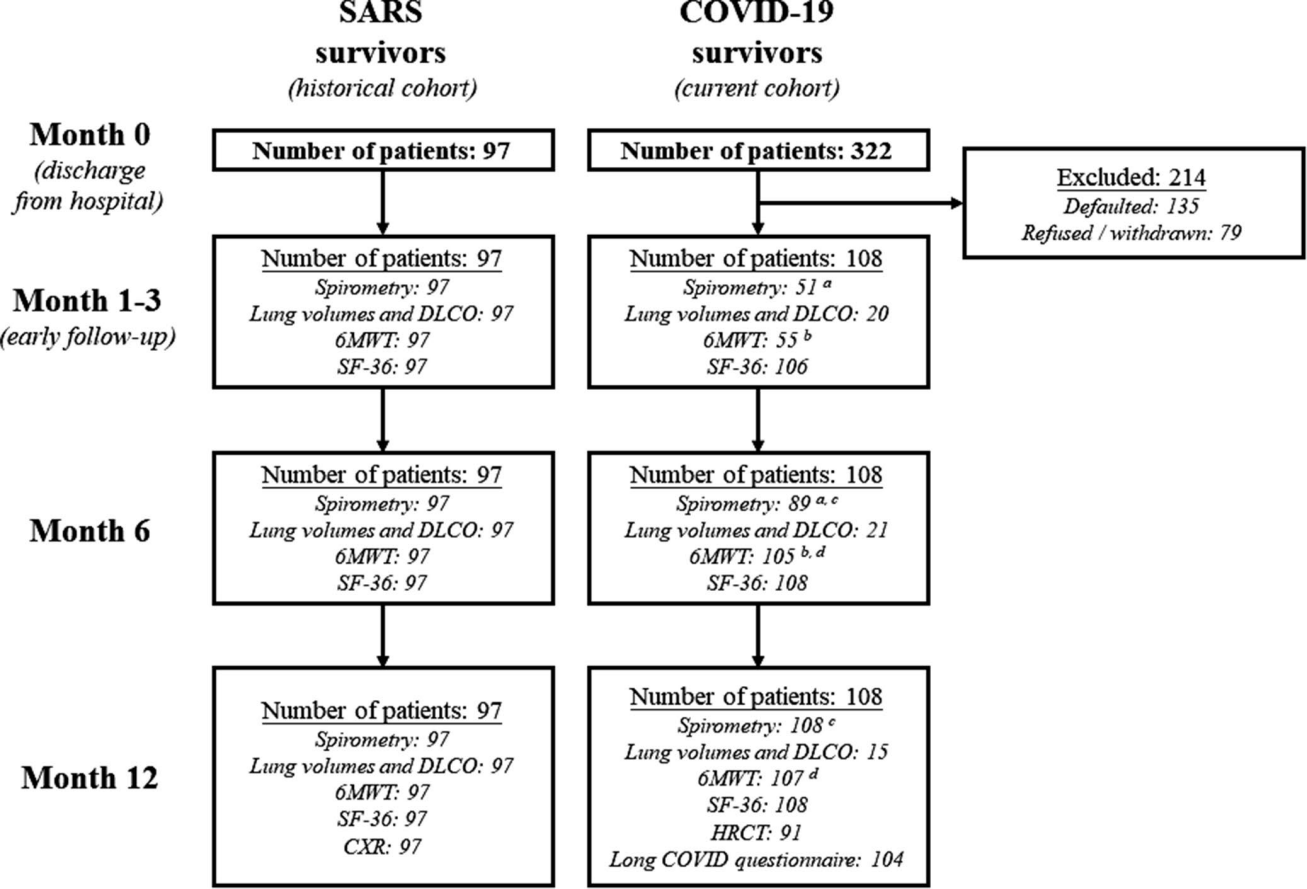
Number of COVID-19 and SARS survivors who had completed the clinical assessments at different time points. ^a^ 41 COVID-19 survivors who performed spirometry both at months 1 to 3 and 6. ^b^ 53 COVID-19 survivors who performed 6-minute walking tests at months 1 to 3 and 6. ^c^ 89 COVID-19 survivors who performed spirometry both at months 6 and 12. ^d^ 104 COVID-19 survivors who performed 6-minute walking tests both at months 6 and 12. *6MWT: 6-minute walking test; COVID-19: coronavirus disease 2019; CXR: chest X-ray; DLCO: diffusion capacity of carbon monoxide; HRCT: high-resolution computed tomography; SARS: severe acute respiratory syndrome; SF-36: Medical Outcomes Study 36-Item Short-Form General Health Survey*

Five hundred and ninety COVID-19 patients were screened and 268 patients refused to join the study. Two hundred and fourteen COVID-19 survivors dropped out from subsequent follow-ups for various reasons, and only 108 of them completed both 6 and 12-month follow-ups. The number of COVID-19 and SARS survivors who underwent various clinical assessments throughout the study is shown in Fig. 1. During the COVID-19 outbreak, especially during the 1-to-3-month follow-up, clinical assessment including full lung function test (lung volumes and DLCO) and 6MWT was heavily compromised due to infection control concerns among the nursing staff. However, the demographics and outcomes of those who had performed spirometry, 6MWT during the 1-to-3-month follow-up, full lung function tests at 6 and 12 months, and those who did not were largely comparable (Tables S1-S2).

Among the 108 COVID-19 survivors, nearly half (52, 48.1%) were men with a mean age of 48.1 ± 16.4 years. None of them had COVID-19 reinfection, and all were unvaccinated with any COVID-19 vaccines before the completion of the study. The circulating strains of SARS-CoV-2 viruses involved in HK were the Wuhan wild type and D614G variant from May to October 2020. However, information on the specific variant was unavailable for individual COVID-19 survivors. The treatment used for COVID-19 during hospitalization is listed in Table S3. Only 2 (1.9%) COVID-19 survivors, but none of the SARS survivors had received lung function tests before the acute infection.

According to the World Health Organization (WHO) classifications of COVID-19 severity, [30] 3 (2.9%), 36 (33.3%), 51 (47.2%), 9 (8.3%) and 9 (8.3%) had asymptomatic, mild, moderate, severe and critical diseases respectively. The diagnosis of pneumonia was defined by clinical signs, symptoms (fever, cough, dyspnoea, tachypnoea) and chest imaging, with its severity based on the occurrence of haemodynamic and respiratory compromise. The disease severity of COVID-19 subjects who were either excluded from screening or subsequent follow-ups was listed in Table S4. Patients who had required intensive care unit (ICU) admission, oxygen, mechanical ventilation (MV) and received corticosteroids were older than those who did not require these treatment modalities. COVID-19 survivors who had required ICU admission or MV had higher %predicted dynamic lung volumes at 6 and 12 months than their counterparts (Tables S5-S6).

### Comparison of COVID-19 survivors, SARS survivors and HK normative data

Compared with the historical cohort of 97 SARS survivors, COVID-19 survivors were older with more comorbidities, similar MV usage but required fewer ICU admissions and oxygen during hospitalization. Sixty-nine (63.9%) and 90 (92.8%) of COVID-19 and SARS survivors had pneumonia respectively. Significantly more SARS survivors underwent pulmonary rehabilitation than COVID-19 survivors. None of the survivors in both groups required long-term oxygen therapy after the index infections (Table 1). Further subgroup analyses based on the level of care are shown in Tables S7-S8.

**Table 1:** Comparison of baseline patient characteristics between COVID-19 and SARS survivors

Patient characteristics	COVID-19 survivors (n = 108)	SARS survivors (n = 97)	Mean difference (95% CI); *p*-value
Age at discharge, years	48.1 ± 16.4	36.1 ± 9.5	12.0 (8.3 to 15.6); <0.001
BMI at 6 months, kg/m^2^	24.3 ± 4.7	23.4 ± 4.1	0.9 (-0.3 to 2.1); 0.161
Male, n (%)	52 (48.1)	39 (40.2)	0.264
Smoking status			
Chronic smokers, n (%)	7 (6.5)	3 (3.1)	0.096
Ex-smokers, n (%)	4 (3.7)	0 (0.0)	
Non-smokers, n (%)	97 (89.8)	94 (96.9)	
Medical comorbidities			
Cardiovascular comorbidities ^a^, n (%)	22 (20.4)	6 (6.2)	0.007
Respiratory comorbidities (excluding OSA) ^b^, n (%)	6 (5.6)	1 (1.0)	0.127
COPD, n (%)	0 (0.0)	1 (1.0)	0.460
Asthma, n (%)	6 (5.6)	0 (0.0)	0.032
Ischemic heart disease, n (%)	0 (0.0)	1 (1.0)	0.460
Stroke, n (%)	1 (0.9)	1 (1.0)	1.000
Diabetes mellitus, n (%)	9 (8.3)	3 (3.1)	0.149
Hypertension, n (%)	16 (14.8)	4 (4.1)	0.017
Malignancy (active and in remission), n (%)	4 (3.7)	1 (1.0)	0.377
Chronic kidney disease, n (%)	2 (1.9)	0 (0.0)	0.501
HBV carrier, n (%)	9 (8.3)	3 (3.1)	0.149
Liver disease (including HBV carrier), n (%)	4 (3.7)	1 (1.0)	0.377
Others, n (%)	45 (41.7)	4 (4.1)	< 0.001
Pneumonia during hospitalization, n (%)	69 (63.9)	90 (92.8)	< 0.001
ICU admission, n (%)	10 (9.3)	31 (32.0)	< 0.001
Length of stay at ICU, days	12.2 ± 6.2	13.5 ± 15.6	-1.3 (-11.5 to 9.0); 0.807
Required oxygen, n (%)	18 (16.7)	41 (42.3)	< 0.001
Required mechanical ventilation, n (%)	9 (8.3)	6 (6.2)	0.602
Received corticosteroids during hospitalization, n (%)	17 (15.7)	61 (62.9)	< 0.001
Length of stay at hospitals, days	20.4 ± 12.2	22.7 ± 14.6	-2.2 (-5.9 to 1.5); 0.234
Received pulmonary rehabilitation after the index infection, n (%)	2 (1.9)	30 (30.9)	< 0.001

^a^ Cardiovascular comorbidities include hypertension, arrhythmia, ischaemic heart disease and heart failure

^b^ Respiratory comorbidties include chronic airway (asthma and COPD) and structural parenchymal diseases

Data are presented as mean ± SD or n (%)

*BMI: body mass index; COPD: chronic obstructive pulmonary disease; COVID-19: coronavirus disease 2019; HBV: hepatitis B virus; ICU: intensive care unit; OSA: obstructive sleep apnoea; SARS: severe acute respiratory syndrome; SD: standard deviation*

**Fig. 2:**
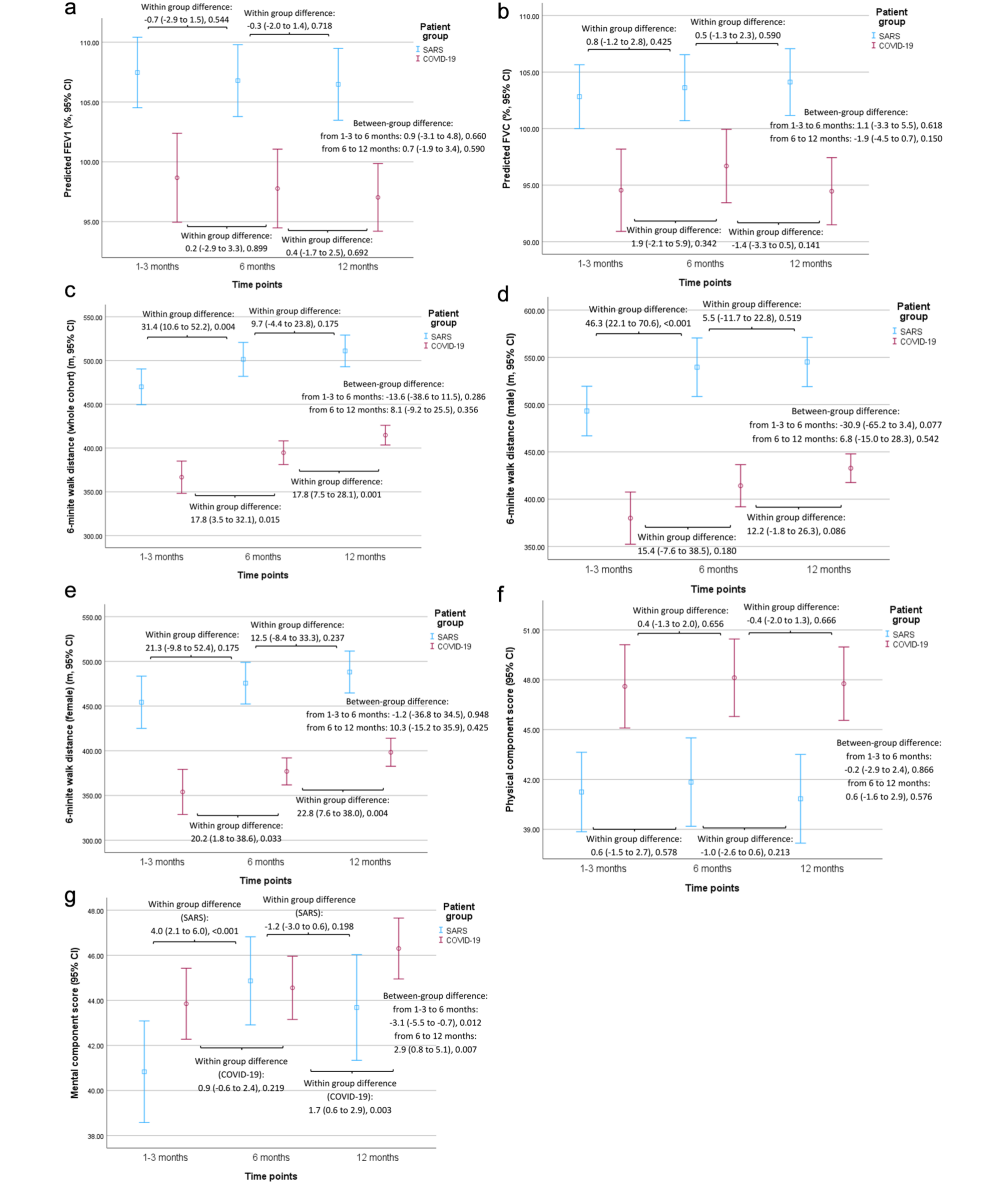
Within-group and between-group differences in serial changes of various physiological parameters and health-related quality of life between COVID-19 and SARS survivors at different time points. Differences are shown in mean ± standard deviation (95% confidence interval) followed by *p*-value. **A**: FEV_1_, **B**: FVC; **C**: 6-minute walking distance of the whole cohort; **D**: 6-minute walking distance of male survivors; **E**: 6-minute walking distance of female survivors; **F**: physical component score; **G**: mental component score. *CI: confidence interval; COVID-19: coronavirus disease 2019; FEV*_*1*_: *forced expiratory volume in 1 s; FVC: forced vital capacity; SARS: severe acute respiratory syndrome*

Both COVID-19 and SARS survivors had %predicted forced expiratory volume in 1 s (FEV_1_), %predicted forced vital capacity (FVC) and static lung volumes within or above the normal range at 6 and 12 months. The %predicted FEV_1_ and %predicted FVC at all 3 visits, and %predicted DLCO at 12 months were lower among COVID-19 survivors (Table 2; Fig. 2A and B). At all 3 visits, the PCS and MCS of both COVID-19 and SARS survivors were generally lower than normal. COVID-19 survivors experienced shorter 6MWD (especially in the younger age groups), but better PCS, MCS (at 1–3 months) and individual SF-36 domains than SARS survivors (Table 3, S9-S12, Fig. 2 C to 2G).

**Table 2:** Comparison of full lung function between COVID-19 and SARS survivors at 1 to 3, 6 and 12 months

	COVID-19 survivors	SARS survivors (n = 97)	Mean difference (95% CI); *p*-value	COVID-19 survivors	SARS survivors (n = 97)	Mean difference (95% CI); *p*-value	COVID-19 survivors	SARS survivors (n = 97)	Mean difference (95% CI); *p*-value
Lung function parameters	1 to 3 months ^a^			6 months ^b^			12 months ^c^		
% predicted FEV_1_, %	98.7 ± 13.2	107.5 ± 14.6	-8.8 (-13.6 to -4.0); <0.001	97.8 ± 15.6	106.8 ± 14.9	-9.0 (-13.4 to -4.6); <0.001	97.0 ± 14.8	106.5 ± 14.9	-9.4 (-13.5 to -5.3); <0.001
% predicted FVC, %	94.6 ± 12.9	102.8 ± 14.0	-8.3 (-12.9 to -3.6); <0.001	96.7 ± 15.4	103.6 ± 14.5	-6.9 (-11.2 to -2.6); 0.002	94.5 ± 15.6	104.1 ± 14.7	-9.7 (-13.8 to -5.5); <0.001
% predicted TLC, %	101.6 ± 18.0	113.7 ± 92.6	-12.1 (-53.4 to 29.2); 0.563	103.4 ± 16.6	106.0 ± 16.7	-2.6 (-10.7 to 5.5); 0.524	102.5 ± 15.7	105.8 ± 16.1	-3.3 (-12.7 to 6.1); 0.484
% predicted VC, %	98.3 ± 17.5	112.4 ± 92.1	-14.1 (-55.1 to 27.0); 0.499	99.8 ± 16.5	103.5 ± 14.9	-3.7 (-11.1 to 3.7); 0.326	99.3 ± 11.5	103.7 ± 15.3	-4.5 (-13.2 to 4.3); 0.313
% predicted RV, %	108.4 ± 36.8	115.6 ± 102.7	-7.1 (-53.4 to 39.1); 0.760	105.5 ± 27.8	110.6 ± 43.2	-5.2 (-20.6 to 10.3); 0.501	115.8 ± 45.2	109.7 ± 38.3	6.1 (-16.8 to 29.0); 0.600
% predicted DLCO, %	83.5 ± 17.6	105.2 ± 93.3	-21.7 (-63.3 to 19.9); 0.304	87.3 ± 11.7	95.5 ± 19.4	-8.2 (-17.1 to 0.8); 0.072	80.2 ± 14.9	91.8 ± 17.7	-11.6 (-21.1 to -2.0); 0.018
% predicted DLCO/V_A_, %	112.3 ± 17.2	116.3 ± 91.7	-4.0 (-44.9 to 36.9); 0.848	115.6 ± 26.9	110.9 ± 14.3	4.7 (-8.2 to 17.6); 0.455	110.0 ± 20.7	114.0 ± 14.5	-4.0 (-12.5 to 4.4); 0.348

^a^ 51 and 20 COVID-19 survivors performed simple spirometry and full lung function measurements at 1 to 3 months respectively

^b^ 89 and 21 COVID-19 survivors performed simple spirometry and full lung function measurements at 6 months respectively

^c^ 108 and 15 COVID-19 survivors performed simple spirometry and full lung function measurements at 12 months respectively

Data are presented as mean ± SD or n (%)

*CI: confidence interval; COVID-19: coronavirus disease 2019; DLCO: diffusion capacity for carbon monoxide; FEV*_*1*_: *forced expiratory volume in 1 s; FVC: forced vital capacity; RV: residual volume; TLC: total lung capacity; SARS: severe acute respiratory syndrome; SD: standard deviation; V*_*A*_: *alveolar volume; VC: vital capacity*

**Table 3:** 6MWD and SF-36 scores among COVID-19 and SARS survivors over 12 months after illness compared with HK normative data

			6 months						12 months			
	Normal		COVID-19 survivors		SARS survivors		**COVID-19 vs. SARS**		COVID-19 survivors	SARS survivors	**COVID-19 vs. SARS**	
	n	mean ± SD	n	mean ± SD; mean Δ (vs. normal) (95% CI)	n	mean ± SD; mean Δ (vs. normal) (95% CI)	Mean Δ (95% CI)	P value	mean ± SD; mean Δ (vs. normal) (95% CI)	mean ± SD; mean Δ (vs. normal) (95% CI)	Mean Δ (95% CI)	P value
6MWD ^a^												
Wole cohort	N/A		104	397.4 ± 64.5	97	501.3 ± 96.2	-104.0 (-126.9 to -81.0)	< 0.001	415.2 ± 58.4	511.0 ± 89.8	-95.8 (-117.1 to -74.6)	< 0.001
Male	N/A		49	420.3 ± 66.4	39	539.5 ± 95.5	-119.3 (-155.2 to -83.3)	< 0.001	432.5 ± 54.3	545.1 ± 80.2	-112.6 (-142.6 to -82.6)	< 0.001
Female	N/A		55	377.0 ± 55.9	58	475.6 ± 88.5	-98.7 (-126.1 to -71.2)	< 0.001	399.7 ± 58.1	488.1 ± 89.2	-88.3 (-116.3 to -60.4)	< 0.001
SF-36 (PCS)												
Whole cohort	2410	52.8 ± 7.3	108	48.1 ± 12.2; -4.7 (-7.1, -2.4) ^b^	97	41.8 ± 13.2; -11.0 (-13.7, -8.3) ^b^	6.3 (2.8 to 9.8)	0.001	47.8 ± 11.6; -5.1 (-7.3 to -2.8) ^b^	40.8 ± 13.2; -6.3 (-8.9 to -3.8) ^b^	6.9 (1.7 to 3.5)	< 0.001
SF-36 (MCS)												
Whole cohort	2410	47.2 ± 9.6	108	44.6 ± 7.4; -2.6 (-4.1, -1.2) ^b^	97	44.9 ± 9.7; -2.3 (-4.3, -0.4) ^d^	-0.3 (-2.7 to 2.0)	0.796	46.3 ± 7.1; -6.5 (-8.0 to -5.0) ^b^	43.7 ± 11.6; -3.5 (-5.9 to -1.1) ^c^	2.6 (1.4 to -0.1)	0.057

^a^ a comparison on 6MWD between the whole cohort, male and female survivors with normal ranges of the population, as the normal ranges of 6MWD are age-specific

^b^ denotes p < 0.001, ^c^ p < 0.01, ^d^ <0.05 between COVID-19 or SARS survivors, and Hong Kong normative data

For analysis of the whole cohort, male and female COVID-19 survivors, only 104 of them who performed a 6-minute walking test at both months 6 and 12 were included in this analysis

*6MWD: 6-minute walking distance; CI: confidence interval; COVID-19: coronavirus disease 2019; HK: Hong Kong; N/A: not applicable; SARS: severe acute respiratory syndrome; SD: standard deviation; SF-36: Medical Outcomes Study 36-Item Short-Form General Health Survey*

### Residual radiological abnormalities on HRCT

Ninety-one COVID-19 survivors underwent HRCT at 12 months, and 40 (44.0%) had radiological abnormalities. Twenty-three (25.3%) patients had parenchymal bands, 13 (14.3%) had pleural thickening, 12 (13.2%) had interlobular lines, 12 (13.2%) had GGO, 7 (7.7%) had bronchiectasis, 4 (4.4%) had mosaic attenuation and 2 (2.2%) had emphysema. None had consolidation, honeycombing, pleural effusion or thoracic lymphadenopathy on the HRCT. The distribution and involvement of GGO, fibrosis and parenchymal changes in each lobe are shown in Figure S1. These radiological abnormalities and their severities were closely related to the need for ICU admission, oxygen, MV and corticosteroids use during hospitalization. The severity scores of involvement by fibrosis and parenchymal changes correlated negatively with 6MWD, but not other lung function parameters at 12 months, while the involvement by GGO was independent of the 6MWD and lung function parameters (Tables S13-S14).

### Within-group differences over 12 months

Both COVID-19 and SARS survivors did not experience significant changes in dynamic lung volumes and PCS between follow-ups. The mean 6MWD of COVID-19 survivors (whole cohort: 3.5 to 32.1 m from 1–3 to 6 month, p = 0.015; 7.5 to 28.1 m, p = 0.001), especially among females (1.8 to 38.6 m from 1–3 to 6 month, p = 0.033; 7.6 to 38.0 m from 6 to 12 month, p = 0.004), increased between the three follow-ups. The MCS of COVID-19 survivors also improved from 6 to 12 months. SARS survivors only had improvement in 6MWD, especially among males, and MCS from 1–3 to 6 months (Tables S15-S17, Fig. 2A and G).

### Between-group differences over 12 months

There was no significant difference in changes in %predicted FEV_1_, %predicted FVC, 6MWD and PCS throughout 12 months between COVID-19 and SARS survivors. Although SARS survivors experienced greater improvement in MCS and some SF-36 domains from 1–3 to 6 months than the COVID-19 survivors, they had a decline in MCS and several other SF-36 domains from 6 to 12 months (Table 4; Fig. 2A and G).

**Table 4:** Comparison of changes in lung function parameters, 6MWD and SF-36 scores over 12 months between COVID-19 and SARS survivors

	From 1–3 to 6 months			From 6 to 12 months		
Parameters	COVID-19 survivors (n = 108)	SARS survivors (n = 97)	Between-group difference; 95% CI; *p-*value	COVID-19 survivors (n = 108)	SARS survivors (n = 97)	Between-group difference; 95% CI; *p-*value
% predicted FEV_1_, % ^a^	0.2 ± 9.8	-0.7 ± 11.0	0.9 (-3.1 to 4.8); 0.660	0.4 ± 9.9	-0.3 ± 8.4	0.7 (-1.9 to 3.4); 0.590
% predicted FVC, % ^a^	1.9 ± 12.6	0.8 ± 9.8	1.1 (-3.3 to 5.5); 0.618	-1.4 ± 9.0	0.5 ± 9.0	-1.9 (-4.5 to 0.7); 0.150
6MWD (whole cohort), m ^b^	17.8 ± 52.3	31.4 ± 103.3	-13.6 (-38.6 to 11.5); 0.286	17.8 ± 52.8	9.7 ± 69.8	8.1 (-9.2 to 25.5); 0.356
6MWD (male), m ^b^	15.4 ± 58.3	46.3 ± 74.8	-30.9 (-65.2 to 3.4); 0.077	12.2 ± 48.8	5.5 ± 53.2	6.8 (-15.0 to 28.3); 0.542
6MWD (female), m ^b^	20.2 ± 46.5	21.3 ± 118.3	-1.2 (-36.8 to 34.5); 0.948	22.8 ± 56.1	12.5 ± 79.4	10.3 (-15.2 to 35.9); 0.425
Physical component score (PCS) ^c^	0.4 ± 8.5	0.6 ± 10.5	-0.2 (-2.9 to 2.4); 0.866	-0.4 ± 8.5	-1.0 ± 7.9	0.6 (-1.6 to 2.9); 0.576
Mental component score (MCS) ^c^	0.9 ± 7.8	4.0 ± 9.7	-3.1 (-5.5 to -0.7); 0.012	1.7 ± 6.0	-1.2 ± 9.0	2.9 (0.8 to 5.1); 0.007
Physical functioning (PF) ^c^	3.0 ± 13.4	1.7 ± 16.1	1.3 (-2.8 to 5.4); 0.541	0.2 ± 14.5	-0.8 ± 12.7	0.9 (-2.8 to 4.7); 0.620
Role limitation due to physical problems (RP) ^c^	4.5 ± 18.7	23.5 ± 45.5	-19.0 (-28.8 to -9.2); <0.001	0.1 ± 16.5	-2.6 ± 37.8	2.7 (-5.5 to 10.9); 0.518
Body pain (BP) ^c^	-3.5 ± 21.2	-2.7 ± 25.4	-0.8 (-7.2 to 5.7); 0.814	-0.1 ± 19.4	-3.0 ± 20.1	2.9 (-2.6 to 8.3); 0.298
General health (GH) ^c^	-1.2 ± 16.8	-1.9 ± 15.4	0.7 (-3.8 to 5.1); 0.771	0.1 ± 15.4	-2.4 ± 11.5	2.4 (-1.3 to 6.2); 0.196
Vitality (VT) ^c^	-1.4 ± 16.3	0.2 ± 11.2	-1.5 (-5.4 to 2.3); 0.439	1.8 ± 15.5	-2.0 ± 11.0	3.8 (0.1 to 7.4); 0.045
Social functioning (SF) ^c^	9.2 ± 28.0	9.0 ± 25.3	0.2 (-7.2 to 7.6); 0.962	5.7 ± 19.5	-3.5 ± 19.2	9.2 (3.8 to 14.5); 0.001
Role limitation due to emotional problems (RE) ^c^	-1.2 ± 24.0	16.8 ± 41.4	-18.0 (-27.5 to -8.5); <0.001	4.2 ± 20.1	-4.8 ± 37.0	9.0 (0.6 to 17.3); 0.035
Mental health (MH) ^c^	-0.9 ± 9.7	1.0 ± 12.4	-2.0 (-5.1 to 1.1); 0.210	-0.3 ± 9.2	0.1 ± 14.3	-0.4 (-3.8, to 2.9); 0.811

^a^ 41 and 89 COVID-19 survivors who performed simple spirometry at both 1 to 3 months and 6 month, and at both months 6 and 12 was included for the comparison respectively. Limited data of full lung function test results precluded meaningful analysis of their serial changes

^b^ 54 and 104 COVID-19 survivors who performed 6MWD measurements at both 1 to 3 months and 6 month, and at both months 6 and 12 were included for the comparison respectively

^c^ 106 and 108 COVID-19 survivors who performed SF-36 assessments at both 1 to 3 months and 6 month, and at both months 6 and 12 were included for the comparison respectively

Data are presented as mean ± SD

*6MWD: 6-minute walking distance; CI: confidence interval; COVID-19: coronavirus disease 2019; FEV*_*1*_: *forced expiratory volume in 1 s; FVC: forced vital capacity; SARS: severe acute respiratory syndrome; SD: standard deviation; SF-36: Medical Outcomes Study 36-Item Short-Form General Health Survey*

Regression analysis retained the presence of respiratory comorbidities as being independently associated with the improvement of %predicted FEV_1_, %predicted FVC and 6MWD, while age ≥ 40 years old was associated with the improvement of MCS in the pooled cohort of COVID-19 and SARS survivors from 6 to 12 months (Table S18).

Multivariate analysis retained the presence of respiratory comorbidities as being independently associated with the improvement of %predicted FEV_1_, %predicted FVC and MCS, whereas the use of oxygen and the presence of parenchymal changes on HRCT at 12 months were associated with the change in 6MWD in COVID-19 survivors from 6 to 12 months (Table S19).

## Discussion

To the best of our knowledge, this is the first study directly comparing various physiological and functional domains between COVID-19 and SARS survivors from a longitudinal perspective. The current cohort included a full spectrum of COVID-19 survivors, ranging from asymptomatic to critical diseases, who were compared with SARS survivors. COVID-19 and SARS survivors reached a similar score on the MCS, but had different paces in the recovery of MCS, with more improvement from 6 to 12 months in the COVID-19 survivors. Despite a difference in the age, baseline comorbid status and disease severity, there was no difference in the change of dynamic lung volumes, 6MWD and PCS between the two groups between 1–3 and 6 months and from 6 to 12 months. When focusing on the changes from 6 to 12 months, the presence of respiratory comorbidities was an essential factor associated with the improvement of various physiological parameters in the pooled cohort of COVID-19 survivors alone. Residual radiological changes were common among COVID-19 survivors, and specific patterns were associated with shorter 6MWD and additional support during hospitalization.

The observations in the study support a similar medium to long-term physiological recovery trajectory after COVID-19 and SARS. The findings of relatively normal dynamic lung volumes with low DLCO [7, 8, 31] and reduced 6MWD [3, 32] were also reported by other prospective cohorts. COVID-19 survivors, who had a less eventful course of disease and comparable baseline smoking status, in general, had consistently inferior age-adjusted dynamic lung volumes than SARS survivors. The commonly found residual radiological changes among SARS [13] and COVID-19 survivors [7, 31–35] may contribute to the persistently lower physiological capacity, especially spirometry performance, [5, 32] gas exchange [7, 13, 32] and 6MWD [7, 32]than normal population. Although the radiological changes are common in our cohort, and were associated with a shorter and slower recovery of 6MWD, we could not confirm its association with dynamic lung volumes (similar to SARS survivors) [13]and DLCO (due to limited data available) [36]. All these suggest the detrimental role of parenchymal involvement as a major determinant of long-term physiological recovery in COVID-19 and SARS survivors [13]. Recent data suggested COVID-19 survivors may have persistent and non-progressive fibrotic changes on CT thorax at a rate of 10–24%, which is comparable to our cohort [5, 37]. It should be realized that factors determining the exercise capacity in COVID-19 survivors could be more complicated and multifactorial. Emerging data suggested that these survivors may have physical deconditioning, as reflected by cardiopulmonary exercise test performance, which is independent of residual radiological and lung function abnormalities, and leading to a reduction in physical capacity [38, 39]. Similar results had been reported for SARS survivors [40].

The positive and independent association of baseline respiratory comorbidities (mainly COPD and asthma in COVID-19 and SARS survivors respectively) and medium to long-term recovery of physiological parameters is a novel finding. This may reflect a slow but progressive recovery of airway status after the initial insult in susceptible patients, which was also observed in patients with COPD after exacerbations in medium-term prospective studies [41]. However, the relatively small number, paucity of baseline lung function test before the index infection and lack of other types of respiratory diseases may preclude the generalization of this result. Although various studies have examined the long-term physiological effects and exercise capacity of COVID-19 survivors, limited data has addressed the physiological recovery trajectory in those with pre-existing chronic airway diseases. A nationwide survey in the United Kingdom found that patients with asthma had increased inhaler use and worse asthma management after COVID-19 infection, but this was not accompanied with any physiological measurements [42].

Despite the older age and inferior physiological capacity, COVID-19 survivors outperformed SARS survivors in most SF-36 domains at different time points and they demonstrated differential paces of recovery. This could be due to a less severe disease course during hospitalization and implied that the HRQoL status cannot be fully translated by the inferior physical status (dynamic lung volumes and 6MWD). The initial slower improvement and later overtaking of MCS in COVID-19 survivors, in comparison with SARS survivors, merits attention. Non-measurable factors, in addition to physiological impairment imposed on individuals, should be considered in the COVID-19 pandemic, as the prolonged period of stringent social distancing measures and city lockdown, might cause a significant psychological and social impact on both normal population and COVID-19 survivors, [43, 44]while all these measures were not implemented for a prolonged period in the SARS era. With time, COVID-19 survivors are expected to recover slowly and adapt to the new social norm, as reflected by the HRQoL recovery trajectory [7].

Our findings have several clinical implications. A structured physiological and psychological rehabilitation should be set up for COVID-19 survivors, especially those with underlying respiratory comorbidities. As 6MWD is closely correlated with residual radiological changes on HRCT, serial 6MWD measurements is a useful surrogate to gauge functional recovery and supplement the radiological changes when HRCT is not readily available. A recent study evaluated 21 participants with long COVID-19 at 7 ± 4 months (baseline) and 14 ± 4 months (follow-up) post-infection and found improvement of DLCO and St George’s Respiratory Questionnaire but these values did not normalize 14 months post-infection [45]. Recent data suggested SARS could lead to permanent lung damage 15 years after the infection, [46] whether there is a similar effect on COVID-19 survivors deserves further exploration.

Several limitations of the study exist. First, the number of full lung function tests and other assessments of COVID-19 survivors in early study period was limited due to tight resources and concern with infection control [31]. However, the comparable baseline characteristics between attendants and non-attendants allows us to generalize the available results and compare them with those of SARS survivors. Similarly, HRCT was not incorporated into the acute care and interim follow-up of COVID-19 survivors, thus lacking important serial radiological abnormalities to correlate the changes of various physiological parameters. Han et al. have shown that fibrotic changes shown in the CT thorax at 6-month follow-up were associated with a higher initial CT score in COVID-19 survivors, baseline disease severity and age [47]. Second, the radiological findings between the two cohorts could not be compared directly, as HRCT was not routinely performed during the follow-up of SARS survivors. However, we believe that HRCT is a better investigation tool in assessing the location and extent of the parenchymal involvement, and a negative correlation between overall reticulation and total parenchymal involvement with DLCO has been demonstrated in SARS survivors [48]. Third, a significant number of COVID survivors defaulted or refused follow-up, and among these, 55.3% of them had pneumonia. This may introduce selection bias in the statistical analysis. Nevertheless, those 108 patients who remained in the study, albeit modest in the sample size, were recruited from 3 different hospitals with a common treatment protocol provided by the HK Hospital Authority. The prospective nature and detailed characterization allowed us to generalize the results in the appropriate clinical context. Fourth, the missing information on specific SARS-CoV-2 variants and the lack of matching disease severity between two groups of survivors may limit the data interpretation.

To conclude, although COVID-19 survivors required less respiratory support during hospitalization with lower dynamic lung volumes (%predicted FEV_1_, %predicted FVC), lower %predicted DLCO and exercise capacity (6MWD) during the recovery phase, they had different recovery trajectories in the mental status but a similar speed of recovery in dynamic lung volumes and exercise capacity when compared with SARS survivors. A significant proportion of COVID-19 survivors had residual radiological changes that were associated with additional support during hospitalization and shorter 6MWD. Long-term and multidimensional monitoring during recovery is advised for COVID-19 survivors.

### Electronic supplementary material

Below is the link to the electronic supplementary material.

Supplementary Material 1

Supplementary Material 2

Supplementary Material 3

## Data Availability

The datasets used and/or analyzed during the current study are available from the corresponding author on reasonable request.

## List of abbreviations

6MWD: 6-minute walking distance
6MWT: 6-minute walking test
BMI: Body mass index
BP: Bodily pain
COPD: Chronic obstructive pulmonary disease
COVID-19: Coronavirus disease 2019
DLCO: Diffusion capacity for carbon monoxide
FEV_1_: Forced expiratory volume in one second
FVC: Forced vital capacity
GGO: Ground-glass opacity
GH: General health
HK: Hong Kong
HRCT: High-resolution computed tomography
HRQoL: Health-related quality of life
ICU: Intensive care unit
MCS: Mental component score
MH: Mental health
MV: Mechanical ventilation
PCS: Physical component score
PF: Physical functioning
RE: Role limitation due to emotional problem
RP: Role limitation due to physical problems
RV: Residual volume
SARS: Severe acute respiratory syndrome
SARS-CoV-1: Severe acute respiratory syndrome coronavirus-1
SARS-CoV-2: Severe acute respiratory syndrome coronavirus-2
SD: Standard deviation
SF: Social functioning
SF-36: Medical Outcomes Study 36-Item Short-Form General Health Survey
TLC: Total lung capacity
V_A_: Alveolar volume
VC: Vital capacity
VT: Vitality
WHO: World Health Organization
