# Supplementary material for: Comparison of the 12-month impact of COVID-19 and SARS on physiological capacity and health-related quality of life
Source: BMC Pulm Med. 2023 Nov 14;23:441. doi: 10.1186/s12890-023-02750-8 (PMC10644631; doi:10.1186/s12890-023-02750-8)
Supplement: Supplementary file 1 — Supplementary Material 1 [file 12890_2023_2750_MOESM1_ESM.docx]

**Supplementary material**

**Title: Comparison of the 12-month impact of COVID-19 and SARS on physiological capacity and health-related quality of life**

**Authors**:

* Ken Ka Pang Chan,^1^ MBChB, * Susanna So Shan Ng,^1^ MBChB, Grace Chung Yan Lui,^2^ MBChB, Ho Sang Leung,^3^ MBBS, Ka Tak Wong,^3^ MBChB, Winnie Chiu Wing Chu,^3^ M.D., Tat On Chan,^4^ M. Phil, Karen Yee Shan Yiu,^1^ MNurs, Eugene Yuk Keung Tso,^5^ MBBS, Kin-Wang To,^1^ MBChB, Jenny Chun Li Ngai,^1^ MBChB, Tommy Wing Ho Yip,^1^ MBChB, Rachel Lai Ping Lo, ^1^ MBChB, Joyce Ka Ching Ng,^1^ MBChB, Fanny Wai San Ko,^1^ M.D., David Shu Cheong Hui,^1^ M.D.

** co-first authors*

**Institutions:**

^1^ Division of Respiratory Medicine, Department of Medicine & Therapeutics, The Chinese University of Hong Kong, Prince of Wales Hospital, Hong Kong

^2^ Division of Infectious Diseases, Department of Medicine & Therapeutics, The Chinese University of Hong Kong, Prince of Wales Hospital, Hong Kong

^3^ Department of Imaging and Interventional Radiology, The Chinese University of Hong Kong, Prince of Wales Hospital, Hong Kong

^4^ The Jockey Club School of Public Health and Primary Care, The Chinese University of Hong Kong

^5^ Department of Medicine, United Christian Hospital, Hong Kong

**Corresponding author full contact details:**

Prof David SC Hui

Mailing address: Department of Medicine and Therapeutics, The Chinese University of Hong Kong, Prince of Wales Hospital, 30-32 Ngan Shing Street, Hong Kong, China.

E-mail address: [dschui@cuhk.edu.hk](mailto:dschui@cuhk.edu.hk)

**Appendix S1:**

**Lung function test:**

The simple spirometry and full lung function tests were performed with the CareFusion SpiroUSB with CareFusion Spirometry PC Software and CareFusion Vmax Encore 229, Sensormedics, USA, respectively. The lung function test was performed according to the standards of the American Thoracic Society [1,2] and the results were compared to the normative data of HK [3]. Static and dynamic lung volumes, and diffusion capacity for carbon monoxide (DLCO) were measured.

**6-minute walking test (6MWT):**

This provides a standardized, objective, integrated assessment of cardiopulmonary and musculoskeletal function that is relevant to daily activities [4,5]. The self-paced 6MWT assesses the sub-maximal level of functional capacity and was applied in a long-term follow-up study of survivors of acute respiratory distress syndrome [6]. The 6-minute walk distances (6MWD) were compared to the normative reference data collected from a population survey of 538 normal healthy subjects in 2004 by the Coordinating Committee in Physiotherapy, Hong Kong Hospital Authority, on two separate days. The 6MWD data stratified into different age groups are available for comparison with the COVID-19 and SARS patients.

**Medical Outcomes Study 36-Item Short-Form General Health Survey (SF-36)**

The SF-36 includes eight multiple-item domains that assess physical functioning (PF), social functioning (SF), role limitation due to physical problems (RP), role limitation due to emotional problems (RE), mental health (MH), bodily pain (BP), vitality (VT), and general health (GH) [7], that can be further grouped into physical and mental component scores (PCS, MCS) [8]. Scores for each aspect can range from 0 to 100, with higher scores indicating better HRQoL. The results were compared to the normative data of HK [9].

**Appendix S2**

**Scanning protocol of high-resolution computed tomography (HRCT):**

Coronavirus disease 2019 (COVID-19) survivors were invited to perform a thin-section HRCT from the lung apices to the diaphragm using a reduced radiation dose protocol at 12 months. Scanning was performed on a 128-detector Computer tomography (CT) unit (Somatom Drive, Siemens, Germany) using spiral acquisition with 0.6mm slice thickness. The subjects were scanned in a supine position during breath-holding at full inspiration with normal radiation dose, and at the prone position with full inspiration with reduced radiation dose. All CT images were reviewed by two radiologists (KTW and HSL), with 26 and 6 years of experience in radiology, respectively using viewing consoles (Syngo.via, Siemens, Germany) according to the Digital Imaging and Communications in Medicine Standard. Images are reviewed independently, with a final decision reached by consensus. The reviewers were aware of the COVID-19 status of the subjects and any previous radiological imaging but were blinded to the clinical information and pulmonary status of the subjects.

For each patient, the CT scans are evaluated for the following characteristics, similar to previously described studies for severe acute respiratory syndrome (SARS) [10,11] and COVID-19 [12-15]. The presence or absence of multiple parameters is evaluated in accordance with the lexicon for HRCT Thorax established by the Fleischner Society of Thoracic Imaging [16]; including (A) consolidation, defined as pulmonary parenchymal attenuation; (B) ground-glass opacity (GGO); (C) interlobular lines, defined by thickened interstitial septum; (D) parenchymal bands, defined as linear opacity extending from the viscera pleura; (E) honeycombing, defined as arrays of closely approximated ring shadows representing clustered cystic air spaces; (F) mosaic attenuation, defined as a patchwork of regions of differing attenuation; (G) presence of nodules; (H) bronchiectasis, defined as bronchial dilatation with respect to accompanying pulmonary arteries; (I) emphysema; (J) pleural thickening; (K) pleural effusion; (L) thoracic lymphadenopathy, defined by any hilar or mediastinal node ≥10mm in short axis diameter. Common incidental parenchymal changes, such as minimal apical fibrosis or plate atelectasis; and parenchymal abnormalities that were known to exist prior to COVID-19 infection, were excluded from grading.

A semi-quantitative severity scoring is used for scoring the severity [17] of abnormalities in each lobe; 0, no involvement; 1, less than 5% involvement; 2, 5%–25% involvement; 3, 26%–49% involvement; 4, 50%–75% involvement; and 5, greater than 75% involvement. This scoring system will be scored in each lobe for (A) all parenchymal abnormalities, (B) combination of consolidation and GGO, and (C) fibrotic changes (combination of interlobular lines, parenchymal band and honeycombing). The distributions consolidation and GGO, as well as fibrotic changes are being recorded as subpleural, bronchocentric or random. For nodules, its distribution is classified as discrete, clustered (more than 5 adjacent nodules on a single CT slice), or diffuse. The size of the largest nodule and their distribution will also be recorded.

**Appendix S3**

**Statistical analysis:**

STROBE guideline was applied. Data were presented as n (%) or mean with standard deviation (SD), as appropriate. Independent *t­*-tests and paired *t*-tests were used to compare the between-group and within-group differences in the changes in lung function parameters, 6MWD and HRQoL over different time points respectively. Further subgroup analysis based on the use of oxygen, mechanical ventilation (MV) and presence of comorbidities between COVID-19 and SARS survivors was performed to take these factors into account for the different disease severity and comorbidities at baseline. Categorical variables were compared using the Chi-square test. Multivariate analysis incorporating the need for oxygen and corticosteroids during hospitalization, age and sex was performed to evaluate the potential determinants of these longitudinal changes. The need for additional support during hospitalization was included as the independent variable in the analysis as there is no shared severity score for both diseases. Spearman correlations were used to analyze associations among radiological changes, lung function tests and 6MWD at 12 months. Patients with missing data were excluded from subsequent analysis. Data were analyzed using Statistical Product and Service Solutions, version 26.0 (IBM Corp., Armonk, NY, USA). All statistical tests were two-tailed. Statistical significance was taken as *p*<0.05.

Table S1: Comparison of clinical data between COVID-19 survivors who performed spirometry and 6MWT, and those who did not during the 1-to-3-month visit.

| Patient characteristics | Spirometry performed  (n = 51) | Spirometry not performed  (n = 57) | Mean difference (95% CI); *p*-value | 6MWT performed  (n = 55) | 6MWT not performed  (n = 53) | Mean difference (95% CI); *p*-value |
| --- | --- | --- | --- | --- | --- | --- |
| Age at discharge, years | 53.6 ± 14.6 | 43.1 ± 16.5 | 10.5 (4.5 to 16.5); <0.001 | 53.0 ± 14.4 | 42.9 ± 16.9 | 10.1 (4.1 to 16.1); 0.001 |
| BMI at 6 months, kg/m^2^ | 23.3 ± 3.8 (n=48) | 26.7 ± 8.5 (n=11) | -3.5 (-9.3 to 2.3); 0.214 | 23.3 ± 3.9 (n=51) | 27.7 ± 9.5 (n=8) | -4.4 (-12.3 to 3.6); 0.236 |
| Male, n (%) | 25 (49.0%) | 27 (47.4%) | 1.000 | 27 (49.1%) | 25 (47.2%) | 0.850 |
| Smoking status | | | | | | |
| Chronic smokers, n (%) | 4 (7.8%) | 3 (5.3%) | 0.586 | 5 (9.1%) | 2 (3.8%) | 0.323 |
| Ex-smokers, n (%) | 1 (2.0%) | 3 (5.3%) |  | 1 (1.8%) | 3 (5.7%) |  |
| Non-smokers, n (%) | 46 (90.2%) | 51 (89.5%) |  | 49 (89.1%) | 48 (90.6%) |  |
| Medical comorbidities | | | | | | |
| COPD, n (%) | 0 (0.0%) | 0 (0.0%) | --- | 0 (0.0%) | 0 (0.0%) | --- |
| Asthma, n (%) | 3 (5.9%) | 3 (5.3%) | 1.000 | 3 (5.5%) | 3 (5.7%) | 1.000 |
| Ischemic heart disease, n (%) | 0 (0.0%) | 0 (0.0%) | --- | 0 (0.0%) | 0 (0.0%) | --- |
| Stroke, n (%) | 1 (2.0%) | 0 (0.0%) | 0.472 | 1 (1.8%) | 0 (0.0%) | 1.000 |
| Diabetes mellitus, n (%) | 3 (5.9%) | 6 (10.5%) | 0.495 | 5 (9.1%) | 4 (7.5%) | 1.000 |
| Hypertension, n (%) | 9 (17.6%) | 7 (12.3%) | 0.589 | 10 (18.2%) | 6 (11.3%) | 0.419 |
| Malignancy (active and in remission), n (%) | 3 (5.9%) | 1 (1.8%) | 0.342 | 3 (5.5%) | 1 (1.9%) | 0.618 |
| Hyperlipidemia, n (%) | 9 (17.6%) | 9 (15.8%) | 0.803 | 11 (20.0%) | 7 (13.2%) | 0.441 |
| Chronic kidney disease, n (%) | 2 (3.9%) | 0 (0.0%) | 0.221 | 2 (3.6%) | 0 (0.0%) | 0.496 |
| HBV carrier, n (%) | 7 (13.7%) | 2 (3.5%) | 0.081 | 7 (12.7%) | 2 (3.8%) | 0.162 |
| Liver disease (including HBV carrier), n (%) | 2 (3.9%) | 2 (3.5%) | 1.000 | 2 (3.6%) | 2 (3.8%) | 1.000 |
| Others, n (%) | 27 (52.9%) | 18 (31.6%) | 0.032 | 29 (52.7%) | 16 (30.2%) | 0.020 |
| ICU admission, n (%) | 5 (9.8%) | 5 (8.8%) | 1.000 | 6 (10.9%) | 4 (7.5%) | 0.742 |
| Length of stay at ICU, days | 9.8 ± 4.3 | 14.6 ± 7.3 | -4.8 (-13.5 to 3.9); 0.240 | 9.7 ± 3.8 | 16.0 ± 7.6 | -6.3 (-14.6 to 1.9); 0.116 |
| Required oxygen, n (%) | 10 (19.6%) | 8 (14.0%) | 0.453 | 11 (20.0%) | 7 (13.2%) | 0.441 |
| Required mechanical ventilation, n (%) | 4 (7.8%) | 5 (8.8%) | 1.000 | 5 (9.1%) | 4 (7.5%) | 1.000 |
| Received corticosteroids during hospitalisation, n (%) | 12 (23.5%) | 5 (8.8%) | 0.062 | 13 (23.6%) | 4 (7.5%) | 0.033 |
| Length of stay at hospitals, days | 17.4 ± 9.4 | 23.2 ± 13.8 | -5.8 (-10.2 to -1.3); 0.012 | 17.0 ± 9.2 | 24.0 ± 13.9 | -6.9 (-11.4 to -2.4); 0.003 |

Data are presented as mean ± SD or n (%)

*6MWT: 6-minute walking test; BMI: body mass index; CI: confidence interval; COPD: chronic obstructive pulmonary disease; COVID-19: coronavirus disease 2019; HBV: hepatitis B virus; ICU: intensive care unit; SD: standard deviation*

Table S2: Comparison of clinical data between COVID-19 survivors who attended full lung function tests and those who did not attend at 6 and 12 months.

|  | 6 months | | | 12 months | | |
| --- | --- | --- | --- | --- | --- | --- |
| Patient characteristics | Full lung function test attended  (n = 21) | Full lung function test not attended  (n = 87) | Mean difference (95% CI); *p*-value | Full lung function test attended  (n = 15) | Full lung function test not attended  (n = 93) | Mean difference (95% CI); *p*-value |
| Age at discharge, years | 49.8 ± 17.2 | 47.6 ± 16.3 | 2.2 (-5.8 to 10.1); 0.590 | 49.6 ± 16.9 | 47.8 ± 16.4 | 1.8 (-7.3 to 10.9); 0.699 |
| BMI at 6 months, kg/m^2^ | 24.9 ± 6.6 | 24.2 ± 4.1 | 0.7 (-1.6 to 3.0); 0.546 | 25.0 ± 3.7 | 24.5 ± 4.6 | 0.5 (-1.9 to 3.0); 0.664 |
| Male, n (%) | 8 (38.1) | 44 (50.6) | 0.340 | 8 (53.3) | 44 (47.3) | 0.783 |
| Smoking status | | | | | | |
| Chronic smokers, n (%) | 0 (0.0) | 7 (8.0) | 0.228 | 0 (0.0) | 7 (7.5) | 0.064 |
| Ex-smokers, n (%) | 0 (0.0) | 4 (4.6) |  | 2 (13.3) | 2 (2.2) |  |
| Non-smokers, n (%) | 21 (100.0) | 76 (87.4) |  | 13 (86.7) | 84 (90.3) |  |
| Medical comorbidities | | | | | | |
| COPD, n (%) | 0 (0.0) | 0 (0.0) | -- | 0 (0.0) | 0 (0.0) | -- |
| Asthma, n (%) | 3 (14.3) | 3 (3.4) | 0.086 | 0 (0.0) | 6 (6.5) | 0.593 |
| Ischemic heart disease, n (%) | 0 (0.0) | 0 (0.0) | -- | 0 (0.0) | 0 (0.0) | -- |
| Stroke, n (%) | 0 (0.0) | 1 (1.1) | 1.000 | 0 (0.0) | 1 (1.1) | 1.000 |
| Diabetes mellitus, n (%) | 2 (9.5) | 7 (8.1) | 1.000 | 1 (6.7) | 8 (8.6) | 1.000 |
| Hypertension, n (%) | 2 (9.5) | 14 (16.1) | 0.733 | 3 (20.0) | 13 (14.0) | 0.694 |
| Malignancy (active and in remission), n (%) | 0 (0.0) | 4 (4.6) | 1.000 | 0 (0.0) | 4 (4.3) | 1.000 |
| Hyperlipidemia, n (%) | 2 (9.5) | 16 (18.4) | 0.516 | 1 (6.7) | 17 (18.3) | 0.458 |
| Chronic kidney disease, n (%) | 0 (0.0) | 2 (2.3) | 1.000 | 0 (0.0) | 2 (2.2) | 1.000 |
| HBV carrier, n (%) | 0 (0.0) | 9 (10.3) | 0.201 | 0 (0.0) | 9 (9.7) | 0.355 |
| Liver disease (including HBV carrier), n (%) | 0 (0.0) | 4 (4.6) | 1.000 | 0 (0.0) | 4 (4.3) | 1.000 |
| Others, n (%) | 5 (23.8) | 40 (46.0) | 0.085 | 8 (53.3) | 37 (39.8) | 0.401 |
| ICU admission, n (%) | 2 (9.5) | 8 (9.2) | 1.000 | 3 (20.0) | 7 (7.5) | 0.143 |
| Length of stay at ICU, days | 18.5 ± 2.1 | 10.6 ± 5.9 | 7.9 (-2.2 to 18.0); 0.109 | 15.7 ± 9.3 | 10.7 ± 4.5 | 5.0 (-4.7 to 14.6); 0.269 |
| Required oxygen, n (%) | 4 (19.0) | 14 (16.1) | 0.749 | 5 (33.3) | 13 (14.0) | 0.126 |
| Required mechanical ventilation, n (%) | 2 (9.5) | 7 (8.1) | 1.000 | 3 (20.0) | 6 (6.5) | 0.109 |
| Received corticosteroids during hospitalization, n (%) | 2 (9.5) | 15 (17.2) | 0.517 | 3 (20.0) | 14 (15.1) | 0.702 |
| Length of stay at hospitals, days | 24.6 ± 9.3 | 19.4 ± 12.6 | 5.2 (-0.6 to 11.0); 0.080 | 26.9 ± 16.5 | 19.4 ± 11.1 | 7.5 (-1.8 to 16.9); 0.106 |

Data are presented as mean ± SD or n (%)

*BMI: body mass index; CI: confidence interval; COPD: chronic obstructive pulmonary disease; COVID-19: coronavirus disease 2019; HBV: hepatitis B virus; ICU: intensive care unit; SD: standard deviation*

Table S3. Treatment for COVID-19 was received during hospitalization.

|  | Disease severity | | | | |
| --- | --- | --- | --- | --- | --- |
| Drug ^a^ | Asymptomatic  (n = 3) | Mild  (n = 36) | Moderate  (n = 51) | Severe  (n = 9) | Critical  (n = 9) |
| Systemic corticosteroids (n, %) | 0 (0.0) | 0 (0.0) | 6 (11.8) | 4 (44.4) | 8 (88.9) |
| Lopinavir / ritonavir (n, %) | 0 (0.0) | 10 (27.8) | 31 (60.8) | 6 (66.7) | 9 (100.0) |
| Ribavirin (n, %) | 1 (33.3) | 8 (22.2) | 28 (54.9) | 1 (11.1) | 3 (33.3) |
| Interferon (n, %) | 1 (33.3) | 5 (13.9) | 30 (58.8) | 4 (44.4) | 8 (88.9) |
| Remdesivir (n, %) | 0 (0.0) | 0 (0.0) | 2 (3.9) | 2 (22.2) | 3 (33.3) |
| Oseltamivir (n, %) | 0 (0.0) | 1 (2.8) | 2 (3.9) | 1 (11.1) | 1 (11.1) |
| Hydroxychloroquine (n, %) | 0 (0.0) | 0 (0.0) | 1 (2.0) | 0 (0.0) | 1 (11.1) |
| Others (n, %) | 0 (0.0) | 0 (0.0) | 0 (0.0) | 0 (0.0) | 2 (22.2) ^b^ |

^a^ Patients may receive more than one treatment during the same hospitalization episode.

^b^ 1 patient received tocilizumab and 1 patient received favipiravir.

Table S4. Disease severity of COVID-19 subjects who were either excluded from screening or subsequent follow-ups.

| Disease severity | Subject number  (Total = 482) |
| --- | --- |
| Asymptomatic, n (%) | 47 (9.8) |
| Mild, n (%) | 168 (34.9) |
| Moderate, n (%) | 231 (47.9) |
| Severe, n (%) | 19 (3.9) |
| Critical, n (%) | 17 (3.5) |

*COVID-19: coronavirus disease 2019*

Table S5. Comparison of demographics and lung function parameters at 6 months and 12 months in COVID-19 survivors who (A) required ICU support vs those treated on medical wards, and (B) required oxygen vs those who did not during hospitalisation for COVID-19.

|  | 1. Required ICU support | | | 1. Used oxygen | | |
| --- | --- | --- | --- | --- | --- | --- |
|  | Yes (n=10) | No (n=98) | Mean difference (95% CI); *p*-value | Yes (n=18) | No (n=90) | Mean difference (95% CI); *p-*value |
| Age, years | 62.2 ± 11.9 | 46.6 ± 16.2 | 15.6 (6.7 to 24.5); 0.002 | 62.2 ± 12.0 | 45.2 ± 15.8 | 16.9 (10.2 to 23.6); <0.001 |
| Male, n (%) | 7 (70.0) | 45 (45.9) | *p*=0.191 | 11 (61.1) | 41 (45.6) | *p*=0.303 |
| BMI at 6 months, kg/m^2^ | 25.7 ± 2.6 | 24.2 ± 4.8 | 1.6 (-1.5 to 4.6); 0.319 | 25.5 ± 5.7 | 24.1 ± 4.5 | 1.4 (-1.0 to 3.8); 0.239 |
| BMI at 12 months, kg/m^2^ | 26.1 ± 2.6 | 24.4 ± 4.6 | 1.7 (-1.3 to 4.6); 0.259 | 26.0 ± 5.4 | 24.3 ± 4.2 | 1.7 (-0.6 to 4.0); 0.143 |
| FEV_1_ at 6 months, L ^a^ | 2.7 ± 0.6 | 2.6 ± 0.8 | 0.2 (-0.4 to 0.7); 0.526 | 2.5 ± 0.6 | 2.6 ± 0.8 | -0.1 (-0.6 to 0.3); 0.586 |
| % predicted FEV_1_ at 6 months, % ^a^ | 106.6 ± 14.8 | 96.9 ± 15.5 | 9.7 (-1.7 to 21.0); 0.095 | 104.3 ± 16.0 | 96.6 ± 15.3 | 7.7 (-1.2 to 16.7); 0.088 |
| FVC at 6 months, L ^a^ | 3.3 ± 0.6 | 3.1 ± 0.8 | 0.2 (-0.4 to 0.8); 0.575 | 3.0 ± 0.6 | 3.2 ± 0.8 | -0.1 (-0.6 to 0.3); 0.592 |
| % predicted FVC at 6 months, % ^a^ | 99.0 ± 14.1 | 96.5 ± 15.6 | 2.5 (-8.9 to 13.9); 0.659 | 99.7 ± 18.8 | 96.1 ± 14.8 | 3.6 (-5.3 to 12.5); 0.426 |
| FEV_1_ at 12 months, L | 2.7 ± 0.6 | 2.6 ± 0.7 | 0.1 (-0.4 to 0.6); 0.614 | 2.4 ± 0.6 | 2.6 ± 0.8 | -0.2 (-0.5 to 0.2); 0.387 |
| % predicted FEV_1_ at 12 months, % | 107.7 ± 12.0 | 95.9 ± 14.7 | 11.7 (2.2 to 21.3); 0.016 | 102.2 ± 14.2 | 96.0 ± 14.8 | 6.2 (-1.3 to 13.8); 0.103 |
| FVC at 12 months, L | 3.3 ± 0.7 | 3.1 ± 0.9 | 0.2 (-0.4 to 0.8); 0.469 | 3.0 ± 0.7 | 3.1 ± 0.9 | -0.1 (-0.5 to 0.3; 0.685 |
| % predicted FVC at 12 months, % | 101.8 ± 13.5 | 93.7 ± 15.6 | 8.1 (-2.1 to 18.3); 0.117 | 97.8 ± 15.4 | 93.8 ± 15.6 | 4.0 (-4.0 to 11.9); 0.328 |

^a^ 89 COVID-19 survivors performed simple spirometry measurements at 6 months. 8 required and 81 did not require ICU support, while 14 required and 75 did not require oxygen.

Data are presented as mean ± SD or n (%)

*BMI: body mass index; CI: confidence interval; COVID-19: coronavirus disease 2019; FEV_1_: forced expiratory volume in 1 second; FVC: forced vital capacity; ICU: intensive care unit*

Table S6. Comparison of demographics and lung function parameters at 6 months and 12 months in COVID-19 survivors who (A) required MV vs those who did not, and (B) used corticosteroids vs those who did not during hospitalisation for COVID-19.

|  | 1. Required MV | | | 1. Used corticosteroids | | |
| --- | --- | --- | --- | --- | --- | --- |
|  | Yes (n=9) | No (n=99) | Mean difference (95% CI); *p*-value | Yes (n=17) | No (n=91) | Mean difference (95% CI); *p-*value |
| Age, years | 61.4 ± 12.4 | 46.9 ± 16.3 | 14.6 (4.8 to 24.4); 0.008 | 61.7 ± 10.9 | 45.5 ± 16.1 | 16.1 (9.7 to 22.5); <0.001 |
| Male, n (%) | 6 (66.7) | 46 (46.5) | *p*=0.308 | 10 (58.8) | 42 (46.2) | *p*=0.430 |
| BMI at 6 months, kg/m^2^ | 26.1 ± 2.5 | 24.2 ± 4.8 | 1.9 (-1.3 to 5.1); 0.242 | 25.5 ± 2.9 | 24.1 ± 4.9 | 1.4 (-1.0 to 3.9); 0.253 |
| BMI at 12 months, kg/m^2^ | 26.5 ± 2.3 | 24.4 ± 4.6 | 2.1 (-0.9 to 5.2); 0.171 | 25.6 ± 3.2 | 24.4 ± 4.6 | 1.2 (-1.1 to 3.6); 0.297 |
| FEV_1_ at 6 months, L ^a^ | 2.8 ± 0.6 | 2.6 ± 0.8 | 0.3 (-0.3 to 0.9); 0.339 | 2.4 ± 0.7 | 2.6 ± 0.8 | -0.3 (-0.7 to 0.2); 0.217 |
| % predicted FEV_1_ at 6 months, % ^a^ | 109.3 ± 13.6 | 96.8 ± 15.4 | 12.5 (0.5 to 24.5); 0.041 | 100.6 ± 14.3 | 97.2 ± 15.9 | 3.4 (-5.2 to 12.0); 0.429 |
| FVC at 6 months, L ^a^ | 3.4 ± 0.6 | 3.1 ± 0.8 | 0.3 (-0.4 to 0.9); 0.399 | 2.9 ± 0.8 | 3.2 ± 0.8 | -0.3 (-0.7 to 0.1); 0.192 |
| % predicted FVC at 6 months, % ^a^ | 101.1 ± 13.8 | 96.3 ± 15.6 | 4.8 (-7.3 to 16.9); 0.435 | 96.1 ± 15.5 | 96.8 ± 15.5 | -0.8 (-9.3 to 7.7); 0.856 |
| FEV_1_ at 12 months, L | 2.8 ± 0.6 | 2.6 ± 0.7 | 0.2 (-0.3 to 0.7); 0.423 | 2.4 ± 0.7 | 2.6 ± 0.7 | -0.3 (-0.6 to 0.1); 0.186 |
| % predicted FEV_1_ at 12 months, % | 110.1 ± 9.9 | 95.8 ± 14.6 | 14.2 (4.3 to 24.1); 0.005 | 99.9 ± 14.5 | 96.5 ± 14.9 | 3.4 (-4.3 to 11.2); 0.385 |
| FVC at 12 months, L | 3.4 ± 0.7 | 3.1 ± 0.9 | 0.3 (-0.3 to 0.9); 0.299 | 2.9 ± 0.9 | 3.1 ± 0.8 | -0.2 (-0.7 to 0.2); 0.302 |
| % predicted FVC at 12 months, % | 104.4 ± 11.3 | 93.6 ± 15.6 | 10.9 (0.3 to 21.5); 0.044 | 94.6 ± 16.1 | 94.4 ± 15.6 | 0.2 (-8.0 to 8.4); 0.968 |

^a^ 89 COVID-19 survivors performed simple spirometry measurements at 6 months. 7 required and 82 did not require MV, while 16 required and 73 did not receive corticosteroids.

Data are presented as mean ± SD or n (%)

*BMI: body mass index; CI: confidence interval; COVID-19: coronavirus disease 2019; FEV_1_: forced expiratory volume in 1 second; FVC: forced vital capacity; ICU: intensive care unit; MV: mechanical ventilation*

Table S7. Comparison of demographics and lung function parameters at 6 months and 12 months in COVID-19 vs SARS survivors who (A) required ICU support and (B) required oxygen, during hospitalization for the illness.

|  | 1. Required ICU support | | | 1. Used oxygen | | |
| --- | --- | --- | --- | --- | --- | --- |
|  | COVID-19 (n=10) | SARS (n=31) | Mean difference (95% CI); *p-*value | COVID-19 (n=18) | SARS (n=41) | Mean difference (95% CI); *p-*value |
| Age, years | 62.2 ± 11.9 | 38.4 ± 9.8 | 23.8 (16.2 to 31.4); <0.001 | 62.2 ± 12.0 | 36.2 ± 8.8 | 25.9 (20.3 to 31.5); <0.001 |
| Male, n (%) | 7 (70.0) | 17 (54.8) | *p*=0.480 | 11 (61.1) | 21 (51.2) | *p*=0.576 |
| BMI at 6 months, kg/m^2^ | 25.7 ± 2.6 | 24.3 ± 3.8 | 1.5 (-1.2 to 4.1); 0.264 | 25.5 ± 5.7 | 23.7 ± 4.0 | 1.8 (-0.8 to 4.4); 0.172 |
| BMI at 12 months, kg/m^2^ | 26.1 ± 2.6 | 24.6 ± 3.6 | 1.5 (-1.0 to 4.0); 0.236 | 26.0 ± 5.4 | 23.9 ± 3.9 | 2.0 (-0.5 to 4.5); 0.109 |
| FEV_1_ at 6 months, L ^a^ | 2.7 ± 0.6 | 2.8 ± 0.7 | -0.1 (-0.6 to 0.5); 0.816 | 2.5 ± 0.6 | 2.9 ± 0.7 | -0.4 (-0.8 to -0.01); 0.047 |
| % predicted FEV_1_ at 6 months, % ^a^ | 106.6 ± 14.8 | 103.8 ± 12.7 | 2.8 (-7.8 to 13.3); 0.600 | 104.3 ± 16.0 | 107.3 ± 12.9 | -3.0 (-11.5 to 5.6); 0.485 |
| FVC at 6 months, L ^a^ | 3.3 ± 0.6 | 3.3 ± 0.9 | -0.002 (-0.7 to 0.7); 0.995 | 3.0 ± 0.6 | 3.5 ± 0.9 | -0.4 (-0.9 to 0.1); 0.101 |
| % predicted FVC at 6 months, % ^a^ | 99.0 ± 14.1 | 98.6 ± 15.7 | 0.4 (-12.0 to 12.8); 0.950 | 99.7 ± 18.8 | 103.4 ± 14.6 | -3.7 (-13.5 to 6.0); 0.448 |
| FEV_1_ at 12 months, L | 2.7 ± 0.6 | 2.8 ± 0.6 | -0.1 (-0.6 to 0.4); 0.627 | 2.4 ± 0.6 | 2.9 ± 0.7 | -0.5 (-0.8 to -0.1); 0.016 |
| % predicted FEV_1_ at 12 months, % | 107.7 ± 12.0 | 103.8 ± 11.5 | 3.8 (-4.7 to 12.4); 0.370 | 102.2 ± 14.2 | 106.0 ± 12.5 | -3.8 (-11.2 to 3.5); 0.303 |
| FVC at 12 months, L | 3.3 ± 0.7 | 3.3 ± 0.8 | -0.01 (-0.6 to 0.6); 0.969 | 3.0 ± 0.7 | 3.4 ± 0.9 | -0.4 (-0.9 to 0.1); 0.083 |
| % predicted FVC at 12 months, % | 101.8 ± 13.5 | 98.5 ± 13.6 | 3.3 (-6.7 to 13.3); 0.504 | 97.8 ± 15.4 | 102.5 ± 14.0 | -4.7 (-12.9 to 3.4); 0.250 |

^a^ 89 COVID-19 survivors performed simple spirometry measurements at 6 months. 8 required ICU support while 14 required oxygen.

Data are presented as mean ± SD or n (%)

*BMI: body mass index; CI: confidence interval; COVID-19: coronavirus disease 2019; FEV_1_: forced expiratory volume in 1 second; FVC: forced vital capacity; ICU: intensive care unit; SARS: severe acute respiratory syndrome*

Table S8. Comparison of demographics and lung function parameters at 6 months and 12 months in COVID-19 vs SARS survivors who (A) required MV and (B) used corticosteroids during hospitalization for the illness.

|  | 1. Required MV | | | 1. Used corticosteroids | | |
| --- | --- | --- | --- | --- | --- | --- |
|  | COVID-19 (n=9) | SARS (n=6) | Mean difference (95% CI); *p-*value | COVID-19 (n=17) | SARS (n=61) | Mean difference (95% CI); *p*-value |
| Age, years | 61.4 ± 12.4 | 37.5 ± 10.8 | 23.9 (10.5 to 37.4); 0.002 | 61.7 ± 10.9 | 35.0 ± 9.0 | 26.6 (21.5 to 31.8); <0.001 |
| Male, n (%) | 6 (66.7) | 2 (33.3) | *p*=0.315 | 10 (58.8) | 26 (42.6) | *p*=0.279 |
| BMI at 6 months, kg/m^2^ | 26.1 ± 2.5 | 24.4 ± 2.4 | 1.7 (-1.1 to 4.6); 0.215 | 25.5 ± 2.9 | 22.8 ± 3.7 | 2.7 (0.7 to 4.6); 0.008 |
| BMI at 12 months, kg/m^2^ | 26.5 ± 2.3 | 25.1 ± 2.7 | 1.4 (-1.4 to 4.3); 0.288 | 25.6 ± 3.2 | 23.2 ± 3.6 | 2.4 (0.5 to 4.4); 0.014 |
| FEV_1_ at 6 months, L ^a^ | 2.8 ± 0.6 | 2.5 ± 0.5 | 0.3 (-0.4 to 1.0); 0.370 | 2.4 ± 0.7 | 2.8 ± 0.7 | -0.5 (-0.9 to -0.1); 0.016 |
| % predicted FEV_1_ at 6 months, % ^a^ | 109.3 ± 13.6 | 104.2 ± 8.7 | 5.2 (-9.1 to 19.4); 0.442 | 100.6 ± 14.3 | 107.0 ± 12.7 | -6.5 (-13.8 to 0.9); 0.082 |
| FVC at 6 months, L ^a^ | 3.4 ± 0.6 | 2.9 ± 0.6 | 0.4 (-0.3 to 1.2); 0.205 | 2.9 ± 0.8 | 3.4 ± 0.8 | -0.5 (-0.9 to -0.01); 0.045 |
| % predicted FVC at 6 months, % ^a^ | 101.1 ± 13.8 | 99.0 ± 11.6 | 2.1 (-13.7 to 17.9); 0.776 | 96.1 ± 15.5 | 103.8 ± 13.5 | -7.8 (-15.6 to 0.04); 0.051 |
| FEV_1_ at 12 months, L | 2.8 ± 0.6 | 2.5 ± 0.3 | 0.3 (-0.4 to 0.8); 0.380 | 2.4 ± 0.7 | 2.8 ± 0.7 | -0.4 (-0.8 to -0.1); 0.018 |
| % predicted FEV_1_ at 12 months, % | 110.1 ± 9.9 | 102.8 ± 6.6 | 7.2 (-2.8 to 17.2); 0.143 | 99.9 ± 14.5 | 105.5 ± 13.5 | -5.6 (-13.1 to 1.9); 0.144 |
| FVC at 12 months, L | 3.4 ± 0.7 | 3.0 ± 0.4 | 0.4 (-0.3 to 1.1); 0.201 | 2.9 ± 0.9 | 3.4 ± 0.8 | -0.5 (-0.9 to 0.003; 0.052 |
| % predicted FVC at 12 months, % | 104.4 ± 11.3 | 99.3 ± 10.5 | 5.1 (-7.4 to 17.7); 0.395 | 94.6 ± 16.1 | 103.6 ± 14.7 | -9.0 (-17.2 to -0.9); 0.031 |

^a^ 89 COVID-19 survivors performed simple spirometry measurements at 6 months. 7 required MV while 16 received corticosteroids.

Data are presented as mean ± SD or n (%)

*BMI: body mass index; CI: confidence interval; COVID-19: coronavirus disease 2019; FEV_1_: forced expiratory volume in 1 second; FVC: forced vital capacity; MV: mechanical ventilation; SARS: severe acute respiratory syndrome*

Table S9. 6MWD among COVID-19 and SARS survivors at 1 to 3 months after illness compared with HK normative data.

|  | Normal | | COVID-19 survivors | | SARS survivors | | **COVID-19 vs SARS** | |
| --- | --- | --- | --- | --- | --- | --- | --- | --- |
| Age group | n | mean ± SD | n | mean ± SD; mean Δ (vs normal) (95% CI) ^a^ | n | mean ± SD; mean Δ (vs normal) (95% CI) ^a^ | Mean Δ (95% CI) | P value |
| Wole cohort | N/A | | 55 | 367 ± 68 | 97 | 470 ± 101 | -103 (-134 to -73) | <0.001 |
| Male | N/A | | 27 | 380 ± 70 | 39 | 493 ± 81 | -113 (-152 to -75) | <0.001 |
| Female | N/A | | 28 | 354 ± 65 | 58 | 454 ± 111 | -100 (-145 to -55) | <0.001 |
| 21-30 | | | | | | | | |
| Male | 80 | 651 ± 105 | 5 | 408 ± 56; -243 (-338 to -148) ^b^ | 14 | 493 ± 53; -158 (-195 to -121) ^b^ | -86 (-144 to -27) | 0.007 |
| Female | 85 | 600 ± 84 | 0 | --- | 15 | 454 ± 85; -146 (-193 to -99) ^b^ | --- | --- |
| 31-40 | | | | | | | | |
| Male | 78 | 645 ± 93 | 1 | 433 | 18 | 513 ± 82; -132 (-179 to -85) ^b^ | --- | --- |
| Female | 108 | 606 ± 86 | 6 | 399 ± 45; -207 (-277 to -137) ^b^ | 22 | 504 ± 130; -102 (-162 to -42) ^c^ | -105 (-217 to 7) | 0.065 |
| 41-50 | | | | | | | | |
| Male | 38 | 623 ± 80 | 4 | 382 ± 43; -241 (-324 to -158) ^b^ | 5 | 488 ± 88; -135 (-213 to -57) ^c^ | -105 (-220 to 9) | 0.065 |
| Female | 79 | 541 ± 67 | 5 | 348 ± 76; -193 (-255 to -131) ^b^ | 13 | 395 ± 78; -146 (-187 to -105) ^b^ | -47 (-133 to 40) | 0.269 |
| 51-60 | | | | | | | | |
| Male | 23 | 588 ± 68 | 6 | 395 ± 117; -193 (-321 to -65) ^d^ | 2 | 331 ± 83; -257 (-362 to -152) ^b^ | 64 (-160 to 288) | 0.512 |
| Female | 33 | 534 ± 89 | 9 | 371 ± 39; -163 (-204 to -122) ^b^ | 7 | 405 ± 98; -129 (-205 to -53) ^c^ | -34 (-125 to 58) | 0.415 |
| 61-70 | | | | | | | | |
| Male | 4 | 484 ± 90 | 7 | 358 ± 40; -126 (-277 to 25) | 0 | --- | --- | --- |
| Female | 14 | 432 ± 54 | 7 | 306 ± 76; -126 (-186 to -66) ^b^ | 1 | 492 | --- | --- |

^a^ a comparison on 6MWD between the whole cohort, male and female survivors with normal ranges of the population, as the normal ranges of 6MWD are age-specific.

^b^ denotes p < 0.001, ^c^ p<0.01, ^d^ <0.05 between COVID-19 or SARS survivors, and Hong Kong normative data

For analysis based on age stratification, as HK normative data only covers age groups ranging from 20 to 70 years old, only 50 COVID-19 survivors corresponding to the same age ranges were included in this comparative analysis.

*6MWD: 6-minute walking distance; CI: confidence interval; COVID-19: coronavirus disease 2019; HK: Hong Kong; N/A: not applicable; SARS: severe acute respiratory syndrome; SD: standard deviation*

Table S10. 6MWD among COVID-19 and SARS survivors over 12 months after illness compared with HK normative data.

|  |  | | 6 months | | | | | | 12 months | | | |
| --- | --- | --- | --- | --- | --- | --- | --- | --- | --- | --- | --- | --- |
|  | Normal | | COVID-19 survivors | | SARS survivors | | **COVID-19 vs SARS** | | COVID-19 survivors | SARS survivors | **COVID-19 vs SARS** | |
| Age group | n | mean ± SD | n | mean ± SD;  mean Δ (vs normal) (95% CI) | n | mean ± SD; mean Δ (vs normal) (95% CI) | Mean Δ (95% CI) | P value | mean ± SD; mean Δ (vs normal) (95% CI) | mean ± SD; mean Δ (vs normal) (95% CI) | Mean Δ (95% CI) | P value |
| 21-30 | | | | | | | | | | | | |
| Male | 80 | 651 ± 105 | 11 | 445 ± 43;  -206 (-242 to -171) ^a^ | 14 | 543 ±72;  -108 (-166 to -50) ^a^ | -98  (-149 to -47) | 0.001 | 443 ± 47;  -208 (-246 to -170) ^a^ | 538 ± 39;  - 113 (-145 to -81) ^a^ | -95  (-131 to -59) | <0.001 |
| Female | 85 | 600 ± 84 | 4 | 448 ± 52;  -152 (-237 to -68) ^a^ | 15 | 490 ± 99;  -110 (-158 to -62) ^a^ | -42  (-151 to 66) | 0.421 | 437 ± 78;  -163 (-247 to -78) ^a^ | 516 ± 89;  -84 (-131 to -37) ^a^ | -79  (-182 to 25) | 0.128 |
| 31-40 | | | | | | | | | | | | |
| Male | 78 | 645 ± 93 | 5 | 414 ± 54;  -231 (-315 to -147) ^a^ | 18 | 551 ± 98;  -94 (-143 to -45) ^a^ | -137  (-233 to -41) | 0.007 | 447 ± 60;  -198 (-281 to -114) ^a^ | 561 ± 89;  -84 (-132 to -36) ^a^ | -114  (-202 to -25) | 0.014 |
| Female | 108 | 606 ± 86 | 15 | 376 ± 51;  -230 (-279 to -181) ^a^ | 22 | 507 ± 49;  -99 (-126 to -72) ^a^ | -131  (-165 to -97) | <0.001 | 406 ± 42;  -200 (-236 to -164) ^a^ | 517 ± 55;  -89 (-118 to -60) ^a^ | -111  (-145 to -77) | <0.001 |
| 41-50 | | | | | | | | | | | | |
| Male | 38 | 623 ± 80 | 6 | 410 ± 60;  -213 (-284 to -142) ^a^ | 5 | 544 ± 132;  -79 (-247 to 89) | -134  (-269 to 1) | 0.051 | 430 ± 36;  -193 (-255 to -130) ^a^ | 542 ± 97;  -81 (-160 to -29) ^c^ | -111  (-229 to 7) | 0.060 |
| Female | 79 | 541 ± 67 | 6 | 386 ± 52;  -155 (-210 to -101) ^a^ | 13 | 468 ± 78;  -73 (-114 to -32) ^a^ | -83  (-156 to -9) | 0.031 | 451 ± 70;  -90 (-144 to -35) ^b^ | 467 ± 104;  -74 (-136 to -12) ^c^ | -8  (-107 to 91) | 0.865 |
| 51-60 | | | | | | | | | | | | |
| Male | 23 | 588 ± 68 | 10 | 453 ± 54;  -135 (-179 to -91) ^a^ | 2 | 405 ± 89;  -183 (-289 to -78) ^b^ | 48  (-52 to 149) | 0.310 | 460 ± 39;  -128 (-158 to -99) ^a^ | 459 ± 178;  -129 (-1727 to 1469) | 1  (-1534 to 1536) | 0.996 |
| Female | 33 | 534 ± 89 | 13 | 393 ± 43;  -141 (-173 to -110) ^a^ | 7 | 362 ± 109;  -172 (-250 to -94) ^a^ | 30  (-71 to 132) | 0.506 | 401 ± 38;  -133 (-165 to -102) ^a^ | 401 ± 92;  -133 (-208 to -58) ^b^ | 0  (-86 to 85) | 0.990 |
| 61-70 | | | | | | | | | | | | |
| Male | 4 | 484 ± 90 | 12 | 386 ± 85;  -98 (-150 to -47) ^b^ | N/A | N/A | N/A | N/A | 415 ± 59;  -69 (-120 to -17) ^c^ | N/A | N/A | N/A |
| Female | 14 | 432 ± 54 | 11 | 339 ± 59;  -93 (-127 to -60) ^a^ | N/A | N/A | N/A | N/A | 361 ± 71;  -71 (-104 to -37) ^a^ | N/A | N/A | N/A |

^a^ denotes p < 0.001, ^b^ p<0.01, ^c^ <0.05 between COVID-19 or SARS survivors, and Hong Kong normative data

For analysis of the whole cohort, male and female COVID-19 survivors, only 104 of them who performed a 6-minute walking test at both months 6 and 12 were included in this analysis. For analysis based on age stratification, as HK normative data only covers age groups ranging from 20 to 70 years old, only 93 COVID-19 survivors corresponding to the same age ranges were included in this comparative analysis.

*6MWD: 6-minute walking distance; CI: confidence interval; COVID-19: coronavirus disease 2019; HK: Hong Kong; N/A: not applicable; SARS: severe acute respiratory syndrome; SD: standard deviation*

Table S11. SF-36 scores among COVID-19 and SARS survivors at 1 to 3 months after illness compared with Hong Kong normative data.

| SF-36 domains / age group | Normal | | COVID-19 survivors | | SARS survivors | | **COVID-19 vs SARS** | |
| --- | --- | --- | --- | --- | --- | --- | --- | --- |
|  | n | mean ± SD | N | mean ± SD;  mean Δ (vs normal) (95% CI) | n | mean ± SD; mean Δ (vs normal) (95% CI) | Mean Δ (95% CI) | P value |
| Physical component score (PCS) | | | | | | | | |
| Overall | 2410 | 52.8 ± 7.3 | 106 | 47.6 ± 13.0;  -5.2 (-7.8 to -2.7)^a^ | 97 | 41.2 ± 11.9;  -11.5 (-13.9 to -9.1)^a^ | 6.4 (2.9 to 9.8) | <0.001 |
| Mental component score (MCS) | | | | | | | | |
| Overall | 2410 | 47.2 ± 9.6 | 106 | 43.8 ± 8.2;  -3.3 (-4.9 to -1.7)^a^ | 97 | 40.8 ± 11.2;  -6.4 (-8.7 to -4.1)^a^ | 3.0 (0.3 to 5.8) | 0.031 |
| Physical functioning (PF) | | | | | | | | |
| 18-40 | 1244 | 96.3 ± 6.6 | 38 | 87.4 ± 19.1;  -8.9 (-15.2 to -2.6)^b^ | 69 | 84.1 ± 15.4;  -12.1 (-15.8 to -8.4)^a^ | 3.4 (-3.5 to 10.0) | 0.342 |
| 41-64 | 695 | 90.6 ± 12.4 | 49 | 77.6 ± 20.6;  -13.1 (-19.1 to -7.1)^a^ | 28 | 63.9 ± 23.2;  -26.7 (-35.7 to -17.6)^a^ | 13.6 (3.4 to 23.8) | 0.009 |
| >65 | 369 | 79.2 ± 19.7 | 19 | 73.9 ± 22.3;  -5.2 (-14.4 to 3.9) | -- | -- | -- | -- |
| Role limitation due to physical problems (RP) | | | | | | | | |
| 18-40 | 1244 | 85.6 ± 27.7 | 38 | 80.8 ± 21.0;  -4.8 (-11.9 to 2.2) | 69 | 44.2 ± 40.9;  -41.4 (-51.3 to -31.5)^a^ | 36.6 (24.7 to 48.4) | <0.001 |
| 41-64 | 695 | 81.6 ± 31.7 | 49 | 73.6 ± 24.7;  -8.0 (-15.5 to -0.6)^c^ | 28 | 21.4 ± 31.7;  -60.2 (-72.2 to -48.2)^a^ | 52.2 (38.2 ± 66.2) | <0.001 |
| >65 | 369 | 73.7 ± 37.2 | 19 | 66.4 ± 26.6;  -7.3 (-20.6 to 6.1) | -- | -- | -- | -- |
| Body pain (BP) | | | | | | | | |
| 18-40 | 1244 | 86.4 ± 19.4 | 38 | 85.6 ± 20.0;  -0.8 (-7.1 to 5.5) | 69 | 77.1 ± 23.8;  -9.3 (-15.1 to -3.5)^b^ | 8.5 (-0.5 to 17.5) | 0.065 |
| 41-64 | 695 | 82.6 ± 22.9 | 49 | 76.5 ± 24.9;  -6.1 (-12.8 to 0.6) | 28 | 52.1 ± 26.8;  -30.6 (-39.3 to -21.8)^a^ | 24.5 (12.4 to 36.6) | <0.001 |
| >65 | 369 | 77.4 ± 26.7 | 19 | 77.3 ± 25.7;  -0.1 (-12.5 to 12.2) | -- | -- | -- | -- |
| General health (GH) | | | | | | | | |
| 18-40 | 1244 | 59.5 ± 19.4 | 38 | 64.0 ± 20.1;  4.5 (-1.8 to 10.7) | 69 | 58.0 ± 19.6;  -1.5 (-6.2 to 3.2) | 6.0 (-1.9 to 13.9) | 0.136 |
| 41-64 | 695 | 53.2 ± 20.1 | 49 | 63.4 ± 20.1;  10.2 (4.4 to 16.0)^a^ | 28 | 41.9 to 14.7;  -11.3 (-17.2 to -5.5)^a^ | 21.5 (12.9 to 30.2) | <0.001 |
| >65 | 369 | 49.2 ± 21.2 | 19 | 57.5 ± 25.9;  8.3 (-1.7 to 18.2) | -- | -- | -- | -- |
| Vitality (VT) | | | | | | | | |
| 18-40 | 1244 | 60.2 ± 18.3 | 38 | 62.8 ± 20.6;  2.6 (-3.3 to 8.6) | 69 | 49.4 ± 10.4;  -10.8 (-13.5 to -8.1)^a^ | 13.4 (6.2 to 20.6) | <0.001 |
| 41-64 | 695 | 60.3 ± 18.9 | 49 | 60.7 ± 21.2;  0.4 (-5.1 to 5.9) | 28 | 42.7 ± 10.3;  -17.6 (-21.9 to -13.4)^a^ | 18.0 (10.9 to 25.2) | <0.001 |
| >65 | 369 | 59.9 ± 19.8 | 19 | 54.9 ± 20.9;  -4.9 (-14.1 to 4.3) | -- | -- | -- | -- |
| Social functioning (SF) | | | | | | | | |
| 18-40 | 1244 | 90.3 ± 16.2 | 38 | 69.1 ± 27.5;  -21.2 (-30.3 to -12.1)^a^ | 69 | 66.5 ± 24.4;  -23.8 (-29.7 to -17.8)^a^ | 2.6 (-7.6 to 12.8) | 0.616 |
| 41-64 | 695 | 92.4 ± 16.5 | 49 | 65.3 ± 29.7;  -27.1 (-35.7 to -18.5)^a^ | 28 | 55.8 ± 25.1;  -36.6 (-46.4 to -26.8)^a^ | 9.5 (-3.8 to 22.8) | 0.158 |
| >65 | 369 | 92.1 ± 17.3 | 19 | 65.8 ± 33.0;  -26.3 (-42.3 to -10.3)^b^ | -- | -- | -- | -- |
| Role limitation due to emotional problems (RE) | | | | | | | | |
| 18-40 | 1244 | 67.7 ± 39.4 | 38 | 78.9 ± 23.3;  11.2 (3.3 to 19.2)^b^ | 69 | 62.8 ± 42.2;  -4.9 (-14.5 to 4.6) | 16.1 (3.6 to 28.7) | 0.012 |
| 41-64 | 695 | 75.0 ± 37.0 | 49 | 77.9 ± 23.2;  2.9 (-4.2 to 10.1) | 28 | 23.8 ± 35.0;  -51.2 (-65.1 to -37.2)^a^ | 54.1 (39.2 to 69.0) | <0.001 |
| >65 | 369 | 78.1 ± 37.2 | 19 | 71.9 ± 31.2;  -6.2 (-23.3 to 10.9) | -- | -- | -- | -- |
| Mental health (MH) | | | | | | | | |
| 18-40 | 1244 | 71.8 ± 16.0 | 38 | 57.9 ± 6.5;  -13.9 (-16.2 to -11.6)^a^ | 69 | 67.8 ± 16.3;  -4.0 (-7.8 to -0.2)^c^ | -9.9 (-14.4 to -5.5) | <0.001 |
| 41-64 | 695 | 73.1 ± 17.6 | 49 | 57.4 ± 8.0;  -15.6 (-18.2 to -13.0)^a^ | 28 | 54.0 ± 18.7;  -19.1 (-25.7 to -12.4)^a^ | 3.4 (-4.1 to 11.0) | 0.361 |
| >65 | 369 | 75.7 ± 18.1 | 19 | 61.1 ± 9.2;  -14.6 (-19.14 to -9.9)^a^ | -- | -- | -- | -- |

^a^ denotes p < 0.001, ^b^ p<0.01, ^c^ <0.05 between COVID-19 or SARS survivors, and Hong Kong normative data

*CI: Confidence interval; COVID-19: coronavirus disease 2019; SARS: severe acute respiratory syndrome; SD: standard deviation; SF-36: Medical Outcomes Study 36-Item Short-Form General Health Survey*

Table S12. SF-36 scores among COVID-19 and SARS survivors over 12 months after illness compared with Hong Kong normative data.

| SF-36 domains / age group |  | | 6 months | | | | | | | | | 12 months | | | | | | |
| --- | --- | --- | --- | --- | --- | --- | --- | --- | --- | --- | --- | --- | --- | --- | --- | --- | --- | --- |
|  | Normal | | COVID-19 survivors | | | SARS survivors | | | **COVID-19 vs SARS** | | | COVID-19 survivors | | SARS survivors | | **COVID-19 vs SARS** | | |
|  | n | mean ± SD | n | mean ± SD;  mean Δ (vs normal) (95% CI) | n | | mean ± SD; mean Δ (vs normal) (95% CI) | Mean Δ (95% CI) | | P value | mean ± SD; mean Δ (vs normal) (95% CI) | | mean ± SD; mean Δ (vs normal) (95% CI) | | Mean Δ (95% CI) | | P value |  |
| Physical component score (PCS) | | | | | | | | | | | | | | | | | | |
| Whole cohort | 2410 | 52.8 ± 7.3 | 108 | 48.1 ± 12.2;  -4.7 (-7.1, -2.4) ^a^ | 97 | | 41.8 ± 13.2;  -11.0 (-13.7, -8.3) ^a^ | 6.3 (2.8 to 9.8) | | 0.001 | 47.8 ± 11.6;  -5.1 (-7.3 to -2.8) ^a^ | | 40.8 ± 13.2;  -6.3 (-8.9 to -3.8) ^a^ | | 6.9 (1.7 to 3.5) | | <0.001 |  |
| Mental component score (MCS) | | | | | | | | | | | | | | | | | | |
| Whole cohort | 2410 | 47.2 ± 9.6 | 108 | 44.6 ± 7.4;  -2.6 (-4.1, -1.2) ^a^ | 97 | | 44.9 ± 9.7;  -2.3 (-4.3, -0.4) ^c^ | -0.3 (-2.7 to 2.0) | | 0.796 | 46.3 ± 7.1;  -6.5 (-8.0 to -5.0) ^a^ | | 43.7 ± 11.6;  -3.5 (-5.9 to -1.1) ^b^ | | 2.6 (1.4 to -0.1) | | 0.057 |  |
| Physical functioning (PF) | | | | | | | | | | | | | | | | | | |
| 18-40 | 1244 | 96.3 ± 6.6 | 40 | 90.5 ± 13.7;  -5.8 (-10.2 to -1.3) ^c^ | 69 | | 84.9 ± 15.0;  -11.3 (-14.9 to -7.7) ^a^ | 5.6  (-0.2 to 11.3) | | 0.057 | 90.6 ± 14.1;  -5.6 (-10.2 to -1.1) ^c^ | | 84.5 ± 16.6;  -11.8 (-15.8 to -7.7) ^a^ | | 6.1  (-0.1 to 12.3) | | 0.053 |  |
| 41-64 | 695 | 90.6 ± 12.4 | 49 | 82.0 ± 17.0);  -8.6 (-13.6 to -3.6) ^b^ | 28 | | 67.9 ± 24.9;  -22.8 (-32.4 to -13.1) ^a^ | 14.2  (3.5 to 24.9) | | 0.011 | 81.3 ± 18.4;  -9.3 (-14.7 to -4.0) ^b^ | | 66.3 ± 26.2;  -24.4 (-34.6 to -14.2) ^a^ | | 15.1  (3.7 to 26.4) | | 0.010 |  |
| >65 | 369 | 79.2 ± 19.7 | 19 | 73.2 ± 19.5;  -6.0 (-15.1 to 3.1) | -- | | -- |  | | -- | 75.8 ± 13.8;  -3.4 (-10.3 to 3.5) | | -- | |  | | -- |  |
| Role limitation due to physical problems (RP) | | | | | | | | | | | | | | | | | | |
| 18-40 | 1244 | 85.6 ± 27.7 | 40 | 84.2 ± 20.7;  -1.4 (-8.1 to 5.4) | 69 | | 74.6 ± 38.0;  -11.0 (-20.2 to -1.7) ^c^ | 9.6  (-3.3 to 22.5) | | 0.091 | 84.2 ± 22.1;  -1.4 (-8.6 to 5.9) | | 66.3 ± 40.2;  -19.3 (-29.1 to -9.5) ^a^ | | 17.9  (6.1 to 29.7) | | 0.003 |  |
| 41-64 | 695 | 81.6 ± 31.7 | 49 | 76.3 ± 22.1;  -5.3 (-12.1 to 1.4) | 28 | | 27.7 ± 39.3;  -53.9 (-69.3 to -38.5) ^a^ | 48.6  (32.2 to 64.9) | | <0.001 | 77.8 ± 24.0;  -3.8 (-11.1 to 3.5) | | 39.3 ± 44.3;  -42.3 (-59.7 to -25.0) ^a^ | | 38.5  (20.2 to 56.9) | | <0.001 |  |
| >65 | 369 | 73.7 ± 37.2 | 19 | 73.7 ± 22.9;  -0.03 (-11.6 to 11.5) | -- | | -- |  | | -- | 70.4 ± 16.3;  -3.3 (-11.9 to 5.3) | | -- | |  | | -- |  |
| Body pain (BP) | | | | | | | | | | | | | | | | | | |
| 18-40 | 1244 | 86.4 ± 19.4 | 40 | 83.4 ± 19.8;  -2.9 (-9.1 to 3.2) | 69 | | 71.4 ± 24.7;  -15.0 (-21.0 to -9.0) ^a^ | 12.1  (3.0 to 21.2) | | 0.010 | 84.1 ± 19.7;  -2.2 (-8.4 to 3.9) | | 68.0 ± 23.5;  -18.4 (-24.1 to -12.6) ^a^ | | 16.2  (7.4 to 24.9) | | <0.001 |  |
| 41-64 | 695 | 82.6 ± 22.9 | 49 | 73.2 ± 24.6;  -9.4 (-16.1 to -2.8) ^b^ | 28 | | 56.9 ± 27.8;  -25.8 (-34.5 to -17.0) ^a^ | 16.3  (3.6 to 29.0) | | 0.009 | 70.7 ± 25.2;  -11.9 (-18.6 to -5.2) ^a^ | | 54.8 ± 29.4;  -27.9 (-39.4 to -16.3) ^a^ | | 15.9  (3.3 to 28.9) | | 0.014 |  |
| >65 | 369 | 77.4 ± 26.7 | 19 | 72.6 ± 24.5;  -4.8 (-17.1 to 7.5) | -- | | -- |  | | -- | 76.8 ± 22.2;  -0.6 (-12.8 to 11.7) | | -- | |  | | -- |  |
| General health (GH) | | | | | | | | | | | | | | | | | | |
| 18-40 | 1244 | 59.5 ± 19.4 | 40 | 63.8 ± 20.2;  4.3 (-1.8 to 10.4) | 69 | | 55.3 ± 17.8;  -4.2 (-8.9 to 0.4) | 8.5  (1.1 to 15.9) | | 0.024 | 62.0 ±18.7;  2.4 (-3.7 to 8.5) | | 52.6 ± 17.9;  -6.9 (-11.5 to -2.2) ^b^ | | 9.3  (2.2 to 16.5) | | 0.011 |  |
| 41-64 | 695 | 53.2 ± 20.1 | 49 | 59.2 ± 20.9;  5.9 (0.1 to 11.8) ^c^ | 28 | | 42.0 ± 19.3;  -11.2 (-18.8 to -3.6) ^b^ | 17.1  (7.5 to 26.8) | | 0.001 | 61.5 ± 20.2;  8.2 (2.4 to 14.1) ^b^ | | 40.4 ± 19.4;  -12.8 (-20.4 to -5.2) ^b^ | | 21.1  (11.7 to 30.5) | | <0.001 |  |
| >65 | 369 | 49.2 ± 21.2 | 19 | 62.1 ± 22.6;  12.9 (3.1 to 22.8) ^c^ | -- | | -- |  | | -- | 60.6 ± 19.7;  11.4 (1.6 ± 21.2) ^c^ | | -- | |  | | -- |  |
| Vitality (VT) | | | | | | | | | | | | | | | | | | |
| 18-40 | 1244 | 60.2 ± 18.3 | 40 | 58.8 ± 19.1;  -1.4 (-7.2 to 4.3) | 69 | | 49.8 ± 11.1;  -10.4 (-13.3 to -7.6) ^a^ | 9.0  (2.4 to 15.6) | | 0.009 | 61.3 ± 20.3;  1.1 (-4.7 to 6.8) | | 47.2 ± 13.7;  -13.0 (-16.4 to -9.6) ^a^ | | 14.1  (6.9 to 21.3) | | <0.001 |  |
| 41-64 | 695 | 60.3 ± 18.9 | 49 | 58.3 ± 20.6;  -2.0 (-7.5 to 3.5) | 28 | | 42.3 ± 14.8;  -18.0 (-25.1 to -10.9) ^a^ | 16.0  (7.9 to 24.1) | | <0.001 | 59.9 ± 22.5;  -0.4 (-7.0 to 6.2) | | 42.0 ± 14.6;  -18.4 (-25.4 to -11.3) ^a^ | | 18.0  (9.5 to 26.4) | | <0.001 |  |
| >65 | 369 | 59.9 ± 19.8 | 19 | 60.2 ± 23.2;  0.3 (-8.9 to 9.6) | -- | | -- |  | | -- | 60.9 ± 20.6;  1.0 (-8.2 to 10.2) | | -- | |  | | -- |  |
| Social functioning (SF) | | | | | | | | | | | | | | | | | | |
| 18-40 | 1244 | 90.3 ± 16.2 | 40 | 78.4 ± 22.3;  -11.8 (-19.0 to -4.6) ^b^ | 69 | | 75.7 ± 19.6;  -14.5 (-19.3 to -9.7) ^a^ | 2.7  (-5.4 to 10.8) | | 0.510 | 86.6 ± 16.6;  -3.7 (-8.8 to 1.4) | | 72.6 ± 23.2;  -17.6 (-23.3 to -12.0) ^a^ | | 13.9  (6.3 to 21.5) | | <0.001 |  |
| 41-64 | 695 | 92.4 ± 16.5 | 49 | 74.7 ± 24.4;  -17.7 (-24.8 to -10.6) ^a^ | 28 | | 45.2 ± 46.4;  -29.7 (-47.9 to -11.5) ^b^ | 10.5  (-1.5 to 22.4) | | 0.086 | 77.3 ± 24.2;  -15.1 (-22.2 to -8.1) ^a^ | | 59.8 ± 27.5;  -32.6 (-43.4 to -21.9) ^a^ | | 17.5  (5.5 to 29.5) | | 0.005 |  |
| >65 | 369 | 92.1 ± 17.3 | 19 | 73.0 ± 24.4;  -19.0 (-31.0 to -7.1) ^b^ | -- | | -- |  | | -- | 81.6 ± 22.6;  -10.5 (-21.5 to 0.5) | | -- | |  | | -- |  |
| Role limitation due to emotional problems (RE) | | | | | | | | | | | | | | | | | | |
| 18-40 | 1244 | 67.7 ± 39.4 | 40 | 76.3 ± 23.7;  8.5 (0.6 to 16.4) ^c^ | 69 | | 77.8 ± 32.2;  10.0 (2.0 to 18.1) ^c^ | -1.5  (-12.2 to 9.2) | | 0.777 | 82.3 ± 19.9;  14.6 (7.8 to 21.3) ^a^ | | 69.1 ± 41.0;  1.3 (-8.2 to 10.9) | | 13.2  (1.6 to 24.8) | | 0.026 |  |
| 41-64 | 695 | 75.0 ± 37.0 | 49 | 76.2 ± 21.3;  1.2 (-5.5 to 7.9) | 28 | | 45.2 ± 46.4;  -29.7 (-47.9 to -11.5) ^b^ | 31.0  (12.1 to 49.8) | | 0.001 | 80.1 ± 22.5;  5.1 (-1.9 to 12.1) | | 50.0 ± 44.9;  -25.0 (-39.1 to -10.8) ^b^ | | 30.1  (11.7 to 48.5) | | 0.002 |  |
| >65 | 369 | 78.1 ± 37.2 | 19 | 70.6 ± 31.2;  -7.5 (-24.6 to 9.6) | -- | | -- |  | | -- | 71.5 ± 21.8;  -6.6 (-17.7 to 4.4) | | -- | |  | | -- |  |
| Mental health (MH) | | | | | | | | | | | | | | | | | | |
| 18-40 | 1244 | 71.8 ± 16.0 | 40 | 55.8 ± 6.8;  -16.1 (-18.4 to -13.7) ^a^ | 69 | | 67.2 ± 14.4;  -4.6 (-8.4 to -0.8) ^c^ | -11.5  (-15.6 to -7.4) | | <0.001 | 56.5 ± 7.1;  -15.3 (-17.7 to -12.9) ^a^ | | 66.1 ± 18.6;  -5.7 (-10.2 to -1.1) ^c^ | | -9.6  (-14.6 to -4.7) | | <0.001 |  |
| 41-64 | 695 | 73.1 ± 17.6 | 49 | 58.0 ± 7.4;  -15.1 (-17.6 to -12.6) ^a^ | 28 | | 59 ± 20.2;  -14.1 (-20.8 to -7.4) ^a^ | -1.0  (-9.1 to 7.0) | | 0.795 | 56.4 ± 8.2;  -16.6 (-19.3 to -13.9) ^a^ | | 62.0 ± 19.6;  -11.1 (-17.7 to -4.4) ^b^ | | -5.6  (-13.5 to 2.3) | | 0.161 |  |
| >65 | 369 | 75.7 ± 18.1 | 19 | 58.7 ± 7.1; -15.5 (-17.0 to -14.0) ^a^ | -- | | -- |  | | -- | 59.2 ± 7.3;  -16.5 (-20.4 to -12.5) ^a^ | | -- | |  | | -- |  |

^a^ denotes p < 0.001, ^b^ p<0.01, ^c^ <0.05 between COVID-19 or SARS survivors, and Hong Kong normative data

*CI: Confidence interval; COVID-19: coronavirus disease 2019; SARS: severe acute respiratory syndrome; SD: standard deviation; SF-36: Medical Outcomes Study 36-Item Short-Form General Health Survey*

Table S13. Comparison of radiological abnormalities on HRCT at 12 months in COVID-19 survivors who (A) required ICU support, (B) required oxygen, (C) required MV, and (D) used corticosteroids versus those who did not during hospitalization for COVID-19.

|  | 1. Required ICU support | | |  | 1. Used Oxygen | | |  | 1. Required MV | | |  | 1. Used corticosteroids | | | |
| --- | --- | --- | --- | --- | --- | --- | --- | --- | --- | --- | --- | --- | --- | --- | --- | --- |
| Radiological abnormalities | Yes (n=9) | No (n=82) | *p-value* |  | Yes (n=15) | No (n=76) | *p-value* |  | Yes (n=8) | No (n=83) | *p-value* |  | Yes (n=15) | No (n=76) | *p-value* |  |
| GGO, n (%) ^a^ | 8 (88.9) | 4 (4.9) | <0.001 |  | 9 (60.0) | 3 (3.9) | <0.001 |  | 7 (87.5) | 5 (6.0) | <0.001 |  | 9 (60.0) | 3 (3.9) | <0.001 |  |
| Interlobular lines, n (%) ^a^ | 6 (66.7) | 6 (7.3) | <0.001 |  | 7 (46.7) | 5 (6.6) | <0.001 |  | 5 (62.5) | 7 (8.4) | 0.001 |  | 8 (53.3) | 4 (5.3) | <0.001 |  |
| Parenchymal bands, n (%) ^a^ | 8 (88.9) | 15 (18.3) | <0.001 |  | 12 (80) | 11 (14.5) | <0.001 |  | 8 (100.0) | 15 (18.1) | <0.001 |  | 9 (60.0) | 14 (18.4) | 0.002 |  |
| Mosaic attenuation, n (%) ^a^ | 1 (11.1) | 3 (3.7) | 0.346 |  | 2 (13.3) | 2 (2.6) | 0.125 |  | 1 (12.5) | 3 (3.6) | 0.312 |  | 2 (13.3) | 2 (2.6) | 0.125 |  |
| Nodules, n (%) ^a^ | 2 (22.2) | 10 (12.2) | 0.338 |  | 5 (33.3) | 7 (9.2) | 0.025 |  | 1 (12.5) | 11 (13.3) | 1.000 |  | 4 (26.7) | 8 (10.5) | 0.106 |  |
| Bronchiectasis, n (%) ^a^ | 2 (22.2) | 5 (6.1) | 0.141 |  | 4 (26.7) | 3 (3.9) | 0.013 |  | 2 (25.0) | 5 (6.0) | 0.114 |  | 3 (20.0) | 4 (5.3) | 0.085 |  |
| Emphysema, n (%) ^a^ | 2 (22.2) | 0 (0.0) | 0.009 |  | 2 (13.3) | 0 (0.0) | 0.026 |  | 1 (12.5) | 1 (1.2) | 0.169 |  | 2 (13.3) | 0 (0.0) | 0.026 |  |
| Pleural thickening, n (%) ^a^ | 1 (11.1) | 12 (14.6) | 1.000 |  | 5 (33.3) | 8 (10.5) | 0.036 |  | 1 (12.5) | 12 (14.5) | 1.000 |  | 0 (0.0) | 13 (17.1) | 0.116 |  |
|  | | | | | | | | | | | | | | | | |
| Severity score of involvement by GGO ^b^ | 3.9 ± 2.8 | 0.2 ± 1.2 | <0.001 |  | 2.7 ± 2.9 | 0.2 ± 1.1 | <0.001 |  | 4.1 ± 2.9 | 0.2 ± 1.2 | <0.001 |  | 2.4 ± 2.7 | 0.2 ± 1.3 | <0.001 |  |
| Severity score of involvement by fibrosis ^b^ | 4.7 ± 2.5 | 0.4 ± 1.0 | <0.001 |  | 3.2 ± 2.7 | 0.4 ± 1.0 | <0.001 |  | 5.0 ± 2.5 | 0.4 ± 1.0 | <0.001 |  | 3.1 ± 2.9 | 0.4 ± 1.0 | <0.001 |  |
| Total severity score by parenchymal changes ^b^ | 5.8 ± 3.3 | 0.5 ± 1.3 | <0.001 |  | 4.1 ± 3.4 | 0.4 ± 1.2 | <0.001 |  | 6.3 ± 3.2 | 0.5 ± 1.3 | <0.001 |  | 3.7 ± 3.5 | 0.5 ± 1.4 | <0.001 |  |

^a^ *p*-values were calculated by Fisher’s exact test. ^b^ *p*-values were determined by Mann-Whitney U test.

Data are presented as mean ± SD or n (%)

A semi-quantitative severity scoring is used for scoring the severity (8) of abnormalities in each lobe; 0, no involvement; 1, less than 5% involvement; 2, 5%–25% involvement; 3, 26%–49% involvement; 4, 50%–75% involvement; and 5, greater than 75% involvement. This scoring system will be scored in each lobe for [1] all parenchymal abnormalities, [2] combination of consolidation and GGO, and [3] fibrotic changes (combination of parenchymal bands, interlobular septal thickening and honeycombing).

*COVID-19: coronavirus disease 2019; GGO: ground-glass opacity; HRCT: high resolution computed tomography; ICU: intensive care unit; MV: mechanical ventilation*

Table S14. Spearman correlations of radiological changes at 12 months with lung function test and 6MWD.

|  | 6MWD | % Predicted FEV_1_ | % Predicted FVC | Change in 6MWD (from 6 months) | Change in % predicted FEV_1_ (from 6 months) | Change in % predicted FVC (from 6 months) | % Predicted DLCO |
| --- | --- | --- | --- | --- | --- | --- | --- |
| Severity score of involvement by consolidation/GGO | -0.072 | 0.123 | 0.027 | 0.053 | 0.048 | 0.097 | -0.284 |
| Severity score of involvement by fibrosis | -0.258 ^a^ | 0.124 | 0.003 | 0.019 | 0.017 | 0.086 | -0.389 |
| Total severity score by parenchymal changes | -0.239 ^a^ | 0.136 | 0.031 | 0.034 | -0.009 | 0.109 | -0.378 |

^a^ denotes *p*<0.05

Fibrosis includes parenchymal bands, interlobular septal thickening and honeycombing on HRCT.

*6MWD: six-minute walking distance; DLCO: diffusion capacity for carbon monoxide; FEV_1_: forced expiratory volume in 1 second; FVC: forced vital capacity; HRCT: high-resolution computed tomography; TLC: total lung capacity*

Table S15. Within-group differences in spirometric parameters, 6MWD and SF-36 scores over 12 months in COVID-19 and SARS survivors.

|  | From 1 – 3 to 6 months | | | | From 6 to 12 months | | | |
| --- | --- | --- | --- | --- | --- | --- | --- | --- |
|  | COVID-19 survivors | | SARS survivors  (n = 97) | | COVID-19 survivors | | SARS survivors  (n = 97) | |
|  | Mean difference ± SD | 95% CI, *p-*value  (within group difference) | Mean difference ± SD | 95% CI, p-value  (within group difference) | Mean difference ± SD | 95% CI, p-value  (within group difference) | Mean difference ± SD | 95% CI, *p-*value  (within group difference) |
| % predicted FEV_1_, % | 0.2 ± 9.8 ^a^ | -2.9 to 3.3, 0.899 | -0.7 ± 11.0 | -2.9 to 1.5, 0.544 | 0.4 ± 9.9 ^b^ | -1.7 to 2.5, 0.692 | -0.3 ± 8.4 | -2.0 to 1.4, 0.718 |
| % predicted FVC, % | 1.9 ± 12.6 ^a^ | -2.1 to 5.9, 0.342 | 0.8 ± 9.8 | -1.2 to 2.8, 0.425 | -1.4 ± 9.0 ^b^ | -3.3 to 0.5, 0.141 | 0.5 ± 9.0 | -1.3 to 2.3, 0.590 |

^a^ 41 COVID-19 survivors who performed spirometry at both 1 to 3 months and 6 month were included in this analysis. Limited data of full lung function test results precluded meaningful analysis of their serial changes.

^b^ 89 COVID-19 survivors who performed spirometry both at 6 and 12 months were included in this analysis. Limited data of full lung function test results precluded meaningful analysis of their serial changes.

*CI: confidence interval; COVID-19: coronavirus disease 2019; FEV_1_: forced expiratory volume in 1 second; FVC: forced vital capacity; SARS: severe acute respiratory syndrome; SD: standard deviation*

Table S16. Changes in 6MWD in COVID-19 and SARS survivors stratified by age groups.

|  |  | From 1-3 to 6 months | | | | From 6 to 12 months | | | |
| --- | --- | --- | --- | --- | --- | --- | --- | --- | --- |
|  |  | COVID-19 survivors  (n = 54) ^a^ | | SARS survivors  (n=97) | | COVID-19 survivors  (n =104) ^b^ | | SARS survivors  (n=97) | |
| Gender | Age | Mean difference ± SD (m) | 95% CI, *p-*value (within group difference) | Mean difference ± SD (m) | 95% CI, *p-*value  (within group difference) | Mean difference ± SD (m) | 95% CI, *p-*value (within group difference) | Mean difference ± SD (m) | 95% CI, *p-*value  (within group difference) |
| Whole cohort | All | 17.8 ± 52.3 | 3.5 to 32.1, 0.015 | 31.4 ± 103.3 | 10.6 to 52.2, 0.004 | 17.8 ± 52.8 | 7.5 to 28.1, 0.001 | 9.7 ± 69.8 | -4.4 to 23.8, 0.175 |
| Male | All | 15.4 ± 58.3 | -7.6 to 38.5, 0.180 | 46.3 ± 74.8 | 22.1 to 70.6, <0.001 | 12.2 ± 48.8 | -1.8 to 26.3, 0.086 | 5.5 ± 53.2 | -11.7 to 22.8, 0.519 |
| Female | All | 20.2 ± 46.5 | 1.8 to 38.6, 0.033 | 21.3 ± 118.3 | -9.8 to 52.4, 0.175 | 22.8 ± 56.1 | 7.6 to 38.0, 0.004 | 12.5 ± 79.4 | -8.4 to 33.3, 0.237 |
| Male | 21 – 30 | 20.4 ± 59.5 (n=5) | -53.4 to 94.2; 0.486 | 49.5 ± 84.5 (n=14) | 0.7 to 98.3; 0.047 | -1.3 ± 52.2  (n=11) | -36.3 to 33.8, 0.937 | -4.7 ± 63.6  (n=14) | -41.5 to 32.0, 0.786 |
|  | 31 – 40 | N/A as n=1 | -- | 38.0 ± 79.1 (n=18) | -1.3 to 77.4; 0.057 | 33.6 ± 12.9  (n=5) | 17.6 to 49.6, 0.004 | 10.3 ± 33.9  (n=18) | -6.5 to 27.2, 0.213 |
|  | 41 – 50 | 16.9 ± 94.1 (n=4) | -132.9 to 166.6; 0.744 | 56.4 ± 50.9 (n=5) | -6.4 to 119.3; 0.067 | 20.3 ± 28.5  (n=6) | -9.5 to 50.2, 0.141 | -2.5 ± 71.2  (n=5) | -90.9 to 85.8, 0.941 |
|  | 51 – 60 | 18.8 ± 46.0 (n=6) | -29.4 to 67.1; 0.362 | 73.1 ± 5.9 (n=2) | 19.7 to 126.5; 0.037 | 6.8 ± 57.4  (n=10) | -34.3 to 47.9, 0.717 | 54.4 ± 89.2  (n=2) | -747.4 to 856.2, 0.547 |
|  | 61 – 70 | -1.8 ± 51.8 (n=7) | -49.7 to 46.1; 0.930 | -- | -- | 29.6 ± 53.8  (n=12) | -4.6 to 63.7, 0.083 | N/A | N/A |
| Female | 21 – 30 | -- | -- | 36.6 ± 84.6 (n=15) | -10.3 to 83.4; 0.116 | -10.5 ± 56.3  (n=4) | -100.1 to 79.1, 0.734 | 25.8 ± 84.0  (n=15) | -21.7 to 72.3, 0.255 |
|  | 31 – 40 | -0.6 ± 46.5 (n=6) | -49.4 to 48.2; 0.977 | 2.8 ± 148.4 (n=22) | -63.1 to 68.6; 0.931 | 30.1 ± 52.3  (n=15) | 1.1 to 59.1, 0.043 | 10.2 ± 72.0  (n=22) | -21.7 to 42.1, 0.512 |
|  | 41 – 50 | 44.4 ± 34.7 (n=5) | 1.3 to 87.5; 0.046 | 73.4 ± 51.9 (n=13) | 42.0 to 104.8; <0.001 | 65.7 ± 82.6  (n=6) | -21.0 to 152.4, 0.109 | -8.8 ± 58.0  (n=13) | -43.8 to 26.3, 0.596 |
|  | 51 – 60 | 15.4 ± 50.6 (n=8) | -27.0 to 57.7; 0.419 | -42.6 ± 144.4 (n=7) | -176.2 to 90.9; 0.464 | 8.1 ± 51.2  (n=13) | -22.9 to 39.0, 0.580 | 38.7 ± 125.8  (n=7) | -77.7 to 155.1, 0.447 |
|  | 61 – 70 | 29.0 ± 51.5 (n=7) | -18.6 to 76.6; 0.187 | N/A as n=1 | -- | 22.8 ± 45.5  (n=11) | -7.7 ± 53.4, 0.127 | N/A as n=1 | N/A |

^a^ 54 COVID-19 survivors, of all ages, who performed 6-minute walking test at both 1-3 months and 6 months were included in this analysis.

^b^ 104 COVID-19 survivors who performed 6-minute walking tests at both 6 and 12 months were included in this analysis.

*6MWD: 6-minute walking distance, CI: confidence interval; COVID-19: coronavirus disease 2019; N/A: not applicable; SARS: severe acute respiratory syndrome; SD: standard deviation*

Table S17. Changes in SF-36 scores in COVID-19 and SARS survivors stratified by age groups.

|  |  | From 1-3 to 6 months | | | | From 6 to 12 months | | | |
| --- | --- | --- | --- | --- | --- | --- | --- | --- | --- |
|  |  | COVID-19 survivors (n=106) ^a^ | | SARS survivors (n=97) | | COVID-19 survivors (n=108) ^b^ | | SARS survivors (n=97) | |
| Domains | Age | Mean difference ± SD | 95% CI, *p*-value | Mean difference ± SD | 95% CI, *p-*value | Mean difference ± SD | 95% CI, *p*-value | Mean difference ± SD | 95% CI, *p-*value |
| PCS | All | 0.4 ± 8.5 | -1.3 to 2.0, 0.656 | 0.6 ± 10.5 | -1.5 to 2.7, 0.578 | -0.4 ± 8.5 | -2.0 to 1.3, 0.666 | -1.0 ± 7.9 | -2.6 to 0.6, 0.213 |
| MCS | All | 0.9 ± 7.8 | -0.6 to 2.4, 0.219 | 4.0 ± 9.7 | 2.1 to 6.0, <0.001 | 1.7 ± 6.0 | 0.6 to 2.9, 0.003 | -1.2 ± 9.0 | -3.0 to 0.6, 0.198 |
| PF | 18 – 40 | 2.9 ± 13.7 | -1.6 to 7.4; 0.202 | 0.8 ± 14.9 | -2.8 to 4.4; 0.657 | 0.1 ± 14.6 | -4.5 to 4.8, 0.957 | -0.4 ± 10.4 | -2.9 to 2.1, 0.729 |
|  | 41 – 64 | 4.9 ± 12.8 | 0.8 to 8.2; 0.017 | 3.9 ± 18.9 | -3.4 to 11.2; 0.280 | -0.7 ± 14.5 | -4.9 to 3.4, 0.723 | -1.6 ± 17.4 | -8.4 to 5.1, 0.629 |
|  | ≥ 65 | -0.8 ± 14.6 | -7.8 to 6.2; 0.816 | -- | -- | 2.6 ± 14.7 | -4.4 to 9.7, 0.444 | N/A | N/A |
| RP | 18 – 40 | 5.4 ± 17.7 | -0.4 to 11.2; 0.066 | 30.4 ± 47.5 | 19.0 to 41.9; <0.001 | 0.0 ± 12.7 | -4.1 to 4.1, 1.000 | -8.3 ± 35.0 | -16.7 to 0.1, 0.052 |
|  | 41 – 64 | 2.7 ± 18.9 | -2.7 to 8.1; 0.326 | 6.3 ± 35.1 | -7.4 to 19.9; 0.355 | 1.5 ± 16.2 | -3.1 to 6.1, 0.511 | 11.6 ± 41.1 | -4.3 to 27.5, 0.147 |
|  | ≥ 65 | 7.2 ± 20.9 | -2.8 to 17.3; 0.148 | -- | -- | -3.3 ± 23.7 | -14.7 to 8.1, 0.553 | N/A | N/A |
| BP | 18 – 40 | -3.0 ± 21.5 | -10.1 to 4.0; 0.391 | -5.7 ± 26.2 | -12.0 to 0.5; 0.073 | 0.7 ± 16.4 | -4.5 to 5.9, 0.789 | -3.4 ± 18.4 | -7.8 to 1.0, 0.133 |
|  | 41 – 64 | -3.4 ± 22.9 | -9.9 to 3.2; 0.311 | 4.8 ± 22.3 | -3.8 to 13.4; 0.264 | -2.5 ± 21.8 | -8.7 to 3.8, 0.432 | -2.1 ± 24.0 | -11.4 to 7.2, 0.646 |
|  | ≥ 65 | -4.6 ± 16.5 | -12.6 to 3.3; 0.238 | -- | -- | 4.2 ± 18.8 | -4.9 to 13.3, 0.346 | N/A | N/A |
| GH | 18 – 40 | -0.3 ± 13.7 | -4.8 to 4.2; 0.897 | -2.7 ± 16.6 | -6.7 to 1.3; 0.176 | -1.8 ± 15.0 | -6.6 to 3.0, 0.445 | -2.6 ± 9.8 | -5.0 to -0.3, 0.028 |
|  | 41 – 64 | -4.2 ± 17.3 | -9.2 to 0.7; 0.092 | 0.1 ± 12.3 | -4.6 to 4.9; 0.951 | 2.3 ± 14.4 | -1.9 to 6.4, 0.273 | -1.6 ± 15.2 | -7.5 to 4.3, 0.572 |
|  | ≥ 65 | 4.6 ± 20.1 | -5.1 to 14.3; 0.329 | -- | -- | -1.5 ± 18.3 | -10.4 to 7.3, 0.721 | N/A | N/A |
| VT | 18 – 40 | -3.3 ± 16.3 | -8.6 to 2.1; 0.221 | 0.4 ± 10.9 | -2.3 to 3.0; 0.784 | 2.5 ± 17.0 | -3.0 to 8.0, 0.359 | -2.6 ± 11.0 | -5.3 to 0.03, 0.053 |
|  | 41 – 64 | -2.4 ± 16.4 | -7.1 to 2.3; 0.306 | -0.4 ± 12.1 | -5.0 to 4.3; 0.877 | 1.7 ± 14.6 | -2.5 to 5.9, 0.432 | -0.4 ± 10.9 | -4.6 to 3.9, 0.863 |
|  | ≥ 65 | 5.3 ± 15.1 | -2.0 to 12.5; 0.145 | -- | -- | 0.7 ± 15.2 | -6.7 to 8.0, 0.852 | N/A | N/A |
| SF | 18 – 40 | 9.9 ± 30.1 | -0.02 to 19.8; 0.050 | 9.2 ± 27.2 | 2.7 to 15.8; 0.006 | 8.1 ± 18.2 | 2.3 to 14.0, 0.008 | -3.1 ± 18.6 | -7.6 to 1.4, 0.174 |
|  | 41 – 64 | 9.4 ± 24.9 | 2.3 to 16.6; 0.011 | 8.5 ± 20.4 | 0.6 to 16.4; 0.037 | 2.6 ± 17.9 | -2.6 to 7.7, 0.322 | -4.5 ± 20.8 | -12.5 to 3.6, 0.265 |
|  | ≥ 65 | 7.3 ± 32.4 | -8.4 to 22.8; 0.343 | -- | -- | 8.6 ± 25.4 | -3.7 to 20.8, 0.159 | N/A | N/A |
| RE | 18 – 40 | -0.4 ± 24.0 | -8.3 to 7.4; 0.911 | 15.0 ± 39.8 | 5.4 to 24.5; 0.003 | 6.0 ± 21.7 | -0.9 to 13.0, 0.086 | -8.7 ± 36.0 | -17.3 to -0.05, 0.049 |
|  | 41 – 64 | -1.7 ± 20.3 | -7.5 to 4.1; 0.560 | 21.4 ± 45.6 | 3.8 to 39.1; 0.019 | 3.9 ± 18.9 | -1.5 to 9.4, 0.155 | 4.8 ± 38.2 | -10.0 to 19.6, 0.515 |
|  | ≥ 65 | -1.3 ± 32.9 | -17.2 to 14.5; 0.864 | -- | -- | 0.9 ± 20.2 | -8.9 to 10.6, 0.852 | N/A | N/A |
| MH | 18 – 40 | -2.1 ± 10.3 | -5.5 to 1.3; 0.216 | -0.6 ± 12.8 | -3.7 to 2.5; 0.708 | 0.8 ± 9.1 | -2.2 to 3.7, 0.605 | -1.1 ± 14.9 | -4.7 to 2.5, 0.541 |
|  | 41 – 64 | 0.5 ± 8.9 | -2.0 to 3.1; 0.690 | 5.0 ± 10.4 | 1.0 to 9.0; 0.017 | -1.5 ± 9.5 | -4.3 to 1.2, 0.264 | 3.0 ± 12.4 | -1.8 to 7.8, 0.209 |
|  | ≥ 65 | -2.4 ± 10.3 | -7.3 to 2.6; 0.331 | -- | -- | 0.5 ± 9.0 | -3.8 to 4.8, 0.801 | N/A | N/A |

^a^ 106 COVID-19 survivors who performed SF-36 questionnaire at both 1 to 3 months and 6 month were included in this analysis.

^b^ 108 COVID-19 survivors who performed SF-36 questionnaire at both 6 and 12 months were included in this analysis

*BP: body pain; CI: confidence interval; COVID-19: coronavirus disease 2019; GH: general health; MCS: mental component score; MH: mental health; N/A: not applicable; PCS: physical component score; PF: physical functioning; RE: role limitation due to emotional problems; RP: role limitation due to physical problems; SARS: severe acute respiratory syndrome; SD: standard deviation; SF: social functioning; SF-36: Medical Outcomes Study 36-Item Short-Form General Health Survey; VI: vitality*

Table S18. Multivariate analysis of the factors determining the change in various parameters from 6 to 12 months of COVID-19 and SARS survivors.

|  | FEV_1_ | FVC | 6MWD | PCS | MCS |
| --- | --- | --- | --- | --- | --- |
| Factors | b;  (95% CI);  *p*-value | b;  (95% CI);  *p*-value | b;  (95% CI);  *p*-value | b;  (95% CI);  *p*-value | b;  (95% CI);  *p*-value |
| COVID-19 | -0.5 (-4.0, 5.1); 0.816 | -2.7 (-7.2, -1.9); 0.249 | -1.1 (-30.1, 27.9); 0.939 | 2.0 (-1.9, 5.8); 0.313 | 3.1 (-0.5, 6.7); 0.095 |
| Age ≥ 40 | 3.6 (-0.7, 7.9); 0.097 | 2.4 (-1.8, 6.6); 0.260 | 6.5 (-22.5, 35.4); 0.661 | 0.8 (-3.0, 4.7); 0.669 | 4.3 (0.7, 7.9); 0.020 |
| Female | -0.3 (-3.1, 2.6); 0.856 | -0.9 (-3.7, 1.8); 0.514 | 5.0 (-13.6, 23.6); 0.599 | -0.9 (-3.3, 1.6); 0.483 | -1.8 (-4.2, 0.5); 0.117 |
| BMI ≥ 25 (at 12 months) | 0.9 (-2.0, 3.9); 0.525 | 1.7 (-1.1, 4.6); 0.237 | 9.1 (-9.9, 28.2); 0.348 | -0.9 (-3.4, 1.6); 0.494 | 0.05 (-2.3, 2.4); 0.968 |
| Required oxygen | -1.4 (-6.3, 3.5); 0.580 | -3.4 (-8.2, -1.4); 0.169 | -9.6 (-42.5, 23.3); 0.567 | -2.8 (-7.2, 1.6); 0.206 | -1.9 (-6.0, 2.3); 0.375 |
| Received corticosteroids during hospitalization | -0.9 (-4.9, 3.0); 0.646 | 1.1 (-2.8, 4.9); 0.586 | 0.6 (-25.4, 26.5); 0.967 | 1.7 (-1.8, 5.1); 0.342 | 0.7 (-2.6, 3.9); 0.683 |
| Presence of respiratory comorbidities (excluding OSA) | 12.1 (4.7, 19.5); 0.001 | 8.2 (1.0, 15.5); 0.027 | 55.7 (8.7, 102.6); 0.020 | -2.7 (-8.9, 3.6); 0.402 | 5.0 (-0.8, 10.9); 0.094 |
| Presence of cardiovascular comorbidities | -2.2 (-6.5, 2.1); 0.321 | -3.0 (-7.3, 1.2); 0.158 | -7.0 (-35.5, 21.6); 0.633 | 1.5 (-2.3, 5.2); 0.444 | -0.4 (-3.9, 3.1); 0.827 |
| Residual radiological changes at 12 months (CXR in SARS survivors and HRCT in COVID-19 survivors) | 1.8 (-1.9, 5.6); 0.339 | 1.9 (-1.9, 5.6); 0.325 | -17.4 (-42.6, 7.7); 0.174 | -0.01 (-3.3, 3.3); 0.996 | 0.3 (-2.9, 3.4); 0.871 |
| Interaction term: oxygen user and residual radiological changes at 12 months (CXR in SARS survivors and HRCT in COVID-19 survivors) | -1.0 (-7.0, 5.0); 0.735 | -0.5 (-6.4, 5.3); 0.856 | 22.3 (-17.9, 62.5); 0.276 | 2.6 (-2.7, 7.9); 0.341 | -1.7 (-6.7, 3.3); 0.501 |
| Interaction term: COVID-19 and age ≥ 40 | -4.9 (-11.0, 1.2); 0.117 | -3.0 (-9.0, 3.0); 0.333 | 9.9 (-29.3, 49.1); 0.622 | -1.6 (-6.8, 3.6); 0.549 | -4.1 (-9.0, 0.8); 0.101 |

*6MWD: 6-minute walking distance; BMI: body mass index; CI: confidence interval; COVID-19: coronavirus disease 2019; CXR: chest X-ray; FEV_1_: forced expiratory volume in 1 second; FVC: forced vital capacity; HRCT: high resolution computed tomography; MCS: mental component score; MV: mechanical ventilation; OSA: obstructive sleep apnoea; PCS: physical component score; SARS: severe acute respiratory syndrome; SD: standard deviation; SF-36: Medical Outcomes Study 36-Item Short-Form General Health Survey*

Table S19. Multivariate analysis for factors associated with changes of various parameters in COVID-19 survivors from 6 to 12 months.

|  | % predicted FEV_1_ | | % predicted FVC | | 6MWD | | PCS | | MCS | |
| --- | --- | --- | --- | --- | --- | --- | --- | --- | --- | --- |
|  | b; 95% CI | *p*-value | b; 95% CI | *p*-value | b; 95% CI | *p*-value | b; 95% CI | *p*-value | b; 95% CI | *p*-value |
| Age ≥ 40 | -3.2 (-8.2, 1.8) | 0.211 | -2.6 (-7.2, 1.9) | 0.256 | 16.3 (-6.6, 39.3) | 0.163 | 0.1 (-3.8, 3.9) | 0.978 | -0.3 (-3.1, 2.5) | 0.831 |
| Female sex | -1.5 (-5.8, 2.8) | 0.496 | -3.2 (-7.2, 0.7) | 0.104 | 2.8 (-17.5, 23.0) | 0.789 | -1.5 (-4.9, 1.9) | 0.383 | 1.3 (-1.2, 3.8) | 0.298 |
| BMI (≥ 25.0) at 12 months | -1.8 (-6.1, 2.6) | 0.419 | 0.3 (-3.6, 4.3) | 0.873 | 5.0 (-15.2, 25.1) | 0.629 | -2.0 (-5.3, 1.4) | 0.253 | 1.6 (-0.9, 4.0) | 0.216 |
| Presence of cardiovascular comorbidities | -0.6 (-6.1, 4.8) | 0.818 | -0.5 (-5.4, 4.4) | 0.846 | -4.2 (-30.4, 22.0) | 0.755 | -1.5 (-5.8, 2.9) | 0.511 | -0.9 (-4.1, 2.3) | 0.600 |
| Presence of respiratory comorbidities (excluding OSA) | 15.4 (6.7, 24.1) | 0.001 | 9.8 (1.9, 17.7) | 0.015 | 38.2 (-1.9, 78.2) | 0.062 | -3.1 (-9.9, 3.6) | 0.366 | 5.4 (0.4, 10.4) | 0.033 |
| Required oxygen | -5.9 (-13.5, 1.7) | 0.128 | -4.7 (-11.5, 2.2) | 0.185 | 63.4 (30.0, 96.8) | <0.001 | -2.1 (-3.5, 7.6) | 0.469 | -1.9 (-5.9, 2.2) | 0.371 |
| Received corticosteroids during hospitalization | 5.3 (-1.3, 11.9) | 0.113 | 4.2 (-1.7, 10.2) | 0.164 | -24.9 (-55.9, 6.0) | 0.114 | 2.9 (-2.2, 8.1) | 0.266 | 0.9 (-2.9, 4.7) | 0.635 |
| Presence of parenchymal changes on HRCT at 12 months | 2.9 (-2.1, 8.0) | 0.257 | 3.0 (-1.6, 7.6) | 0.199 | -33.0 (-57.3, -8.8) | 0.008 | -1.5 (-5.5, 2.6) | 0.482 | 1.0 (-1.9, 4.0) | 0.497 |

*6MWD: 6-minute walking distance; BMI: body mass index; CI: confidence interval; COVID-19: coronavirus disease 2019; CXR: chest X-ray; FEV_1_: forced expiratory volume in 1 second; FVC: forced vital capacity; HRCT: high resolution computed tomography; MCS: mental component score; MV: mechanical ventilation; OSA: obstructive sleep apnoea; PCS: physical component score; SD: standard deviation; SF-36: Medical Outcomes Study 36-Item Short-Form General Health Survey*

Figure S1. Distribution and severity of involvement by ground-glass opacity (A), fibrosis (B) and parenchymal changes (C) as demonstrated by HRCT.


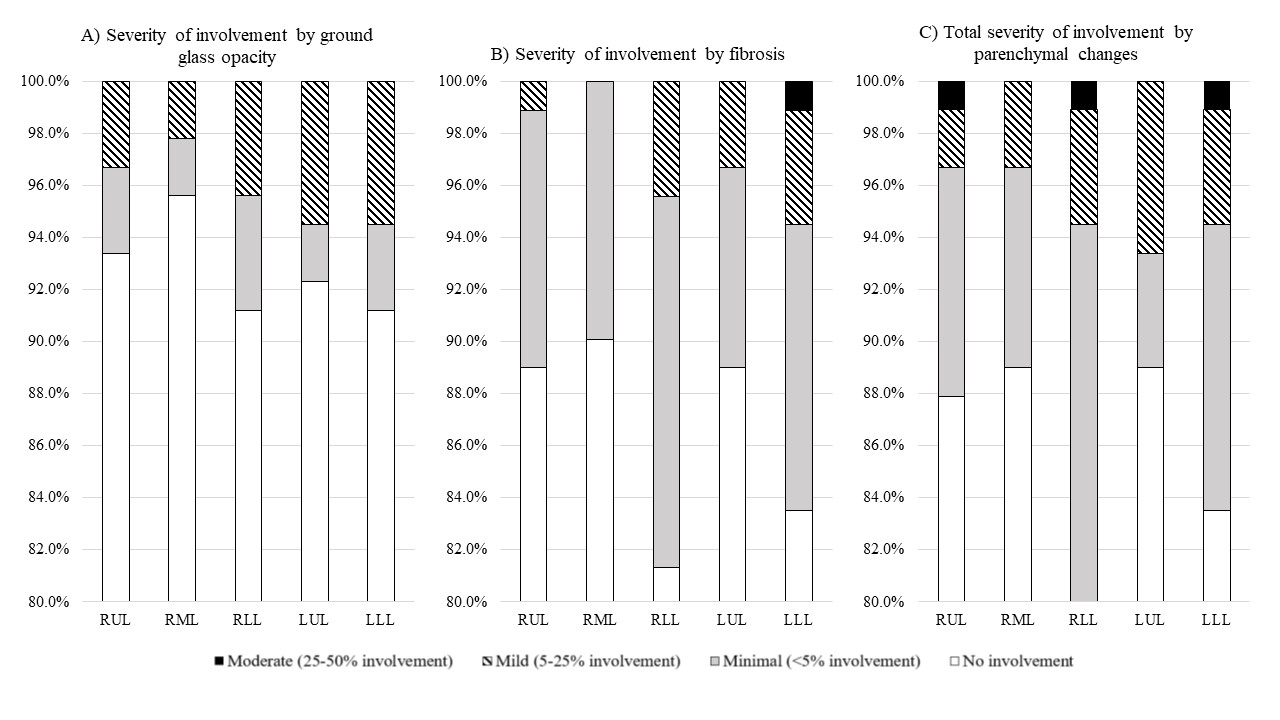


Fibrosis includes parenchymal bands, interlobular septal thickening and honeycombing on HRCT.

*HRCT: high-resolution computed tomography; LLL: left lower lobe; LUL: left upper lobe, RLL: right lower lobe; RML: right middle lobe; RUL: right upper lobe*

References:

1. Standardization of Spirometry, 1994 Update. American Thoracic Society. Am J Respir Crit Care Med. 1995;152(3):1107-1136.
2. Lung function testing: selection of reference values and interpretative strategies. American Thoracic Society. Am Rev Respir Dis. 1991;144(5):1202-1218.
3. Ip MS, Ko FW, Lau AC *et al*; Hong Kong Thoracic Society and American College of Chest Physicians (Hong Kong and Macau Chapter). Updated spirometric reference values for adult Chinese in Hong Kong and implications on clinical utilization. Chest. 2006;129(2):384-392.
4. ATS Committee on Proficiency Standards for Clinical Pulmonary Function Laboratories. ATS statement: guidelines for the six-minute walk test. Am J Respir Crit Care Med. 2002;166(1):111-117.
5. Weisman IM, Zeballos RJ. Clinical exercise testing. Clin Chest Med. 2001;22(4):679-701, viii.
6. Herridge MS, Cheung AM, Tansey CM *et al*; Canadian Critical Care Trials Group. One-year outcomes in survivors of the acute respiratory distress syndrome. N Engl J Med. 2003;348(8):683-693.
7. McHorney CA, Ware JE Jr, Lu JF *et al*. The MOS 36-item Short-Form Health Survey (SF-36): III. Tests of data quality, scaling assumptions, and reliability across diverse patient groups. Med Care. 1994;32(1):40-66.
8. Lam CL, Tse EY, Gandek B *et al*. The SF-36 summary scales were valid, reliable, and equivalent in a Chinese population. J Clin Epidemiol. 2005 Aug;58(8):815-22.
9. Lam CL, Lauder IJ, Lam TP *et al*. Population based norming of the Chinese (HK) version of the SF 36 health survey. Hong Kong Practitioner 1999: 21: 460-470.

Antonio GE, Wong KT, Hui DS *et al*. Thin-section CT in patients with severe acute respiratory syndrome following hospital discharge: preliminary experience. Radiology. 2003;228(3):810-815.

Chu WC, Li AM, Ng AW *et al*. Thin-Section CT 12 Months After the Diagnosis of Severe Acute Respiratory Syndrome in Pediatric Patients. AJR Am J Roentgenol. 2006;186(6):1707-1714.

Chung M, Bernheim A, Mei X *et al*. CT Imaging Features of 2019 Novel Coronavirus (2019-nCoV). Radiology. 2020;295(1):202-207.

Pan F, Ye T, Sun P *et al*. Time Course of Lung Changes at Chest CT during Recovery from Coronavirus Disease 2019 (COVID-19). Radiology. 202;295(3):715-721.

Han X, Fan Y, Alwalid O *et al*. Six-month Follow-up Chest CT Findings after Severe COVID-19 Pneumonia. Radiology. 2021;299(1):E177-E186.

Zhao YM, Shang YM, Song WB *et al*. Follow-up study of the pulmonary function and related physiological characteristics of COVID-19 survivors three months after recovery. EClinicalMedicine. 2020;25:100463.

Hansell DM, Bankier AA, MacMahon H, McLoud TC, Müller NL, Remy J. Fleischner Society: glossary of terms for thoracic imaging. Radiology. 2008;246(3):697-722.

Chang YC, Yu CJ, Chang SC *et al*. Pulmonary sequelae in convalescent patients after severe acute respiratory syndrome: evaluation with thin-section CT. Radiology. 2005;236(3):1067-1075.
