# Supplementary material for: Comparison of the 12-month impact of COVID-19 and SARS on physiological capacity and health-related quality of life
Source: BMC Pulm Med. 2023 Nov 14;23:441. doi: 10.1186/s12890-023-02750-8 (PMC10644631; doi:10.1186/s12890-023-02750-8)
Supplement: Supplementary file 2 — Supplementary Material 2 [file 12890_2023_2750_MOESM2_ESM.docx]

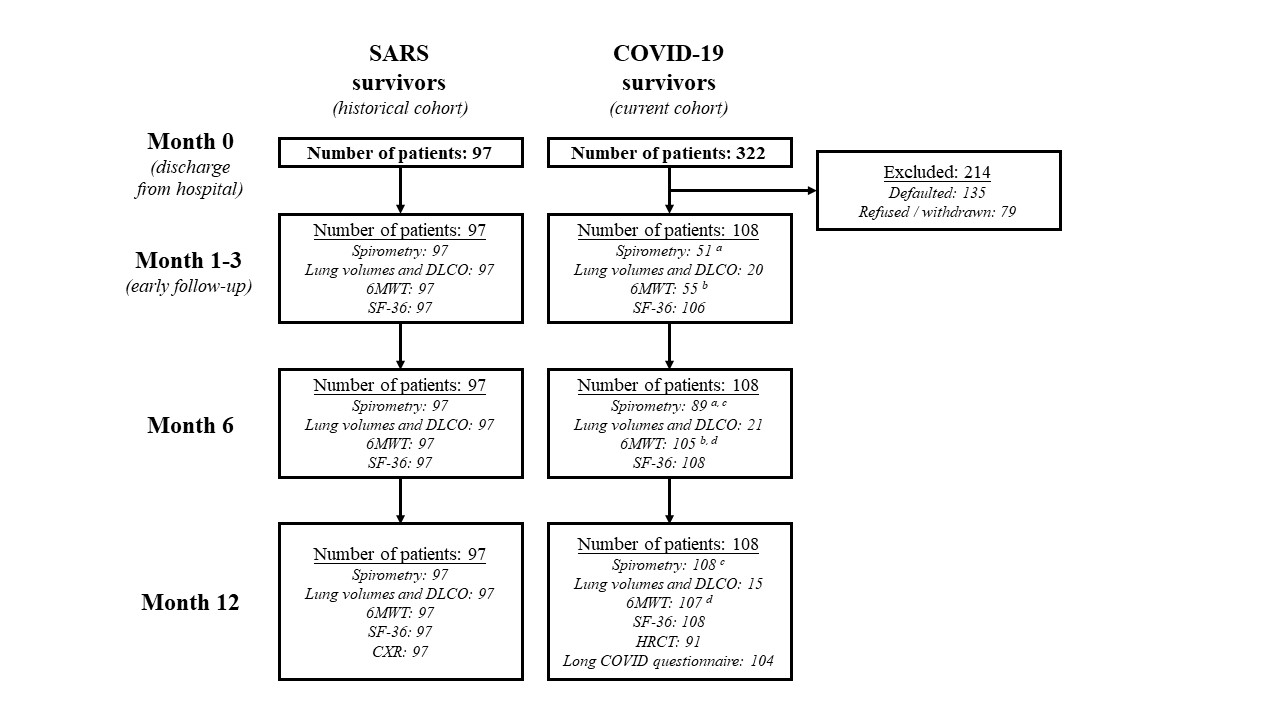


Figure 1. Number of COVID-19 and SARS survivors who had completed the clinical assessments at different time points.

^a^ 41 COVID-19 survivors who performed spirometry both at months 1 to 3 and 6.

^b^ 53 COVID-19 survivors who performed 6-minute walking tests at months 1 to 3 and 6.

^c^ 89 COVID-19 survivors who performed spirometry both at months 6 and 12.

^d^ 104 COVID-19 survivors who performed 6-minute walking tests both at months 6 and 12.

*6MWT: 6-minute walking test; COVID-19: coronavirus disease 2019; CXR: chest X-ray; DLCO: diffusion capacity of carbon monoxide; HRCT: high-resolution computed tomography; SARS: severe acute respiratory syndrome; SF-36: Medical Outcomes Study 36-Item Short-Form General Health Survey*


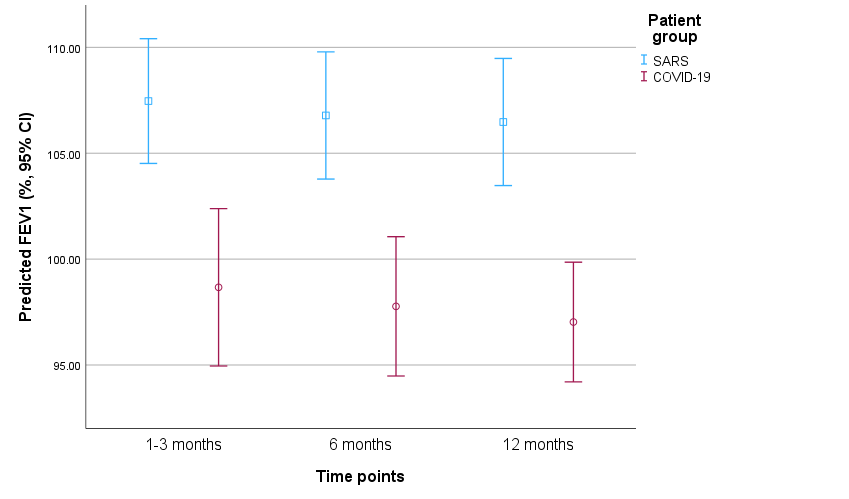


Between-group difference:
from 1-3 to 6 months: 0.9 (-3.1 to 4.8), 0.660
from 6 to 12 months: 0.7 (-1.9 to 3.4), 0.590

Within group difference:
0.4 (-1.7 to 2.5), 0.692

Within group difference:
0.2 (-2.9 to 3.3), 0.899

Within group difference:
-0.7 (-2.9 to 1.5), 0.544

Within group difference:
-0.3 (-2.0 to 1.4), 0.718

Figure 2A. FEV_1_


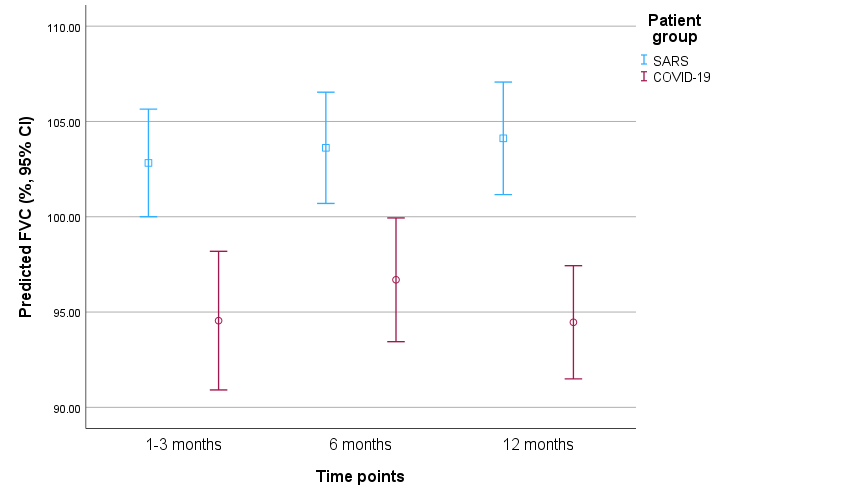


Between-group difference:
from 1-3 to 6 months: 1.1 (-3.3 to 5.5), 0.618
from 6 to 12 months: -1.9 (-4.5 to 0.7), 0.150

Within group difference:
-1.4 (-3.3 to 0.5), 0.141

Within group difference:
1.9 (-2.1 to 5.9), 0.342

Within group difference:
0.5 (-1.3 to 2.3), 0.590

Within group difference:
0.8 (-1.2 to 2.8), 0.425

Figure 2B. FVC


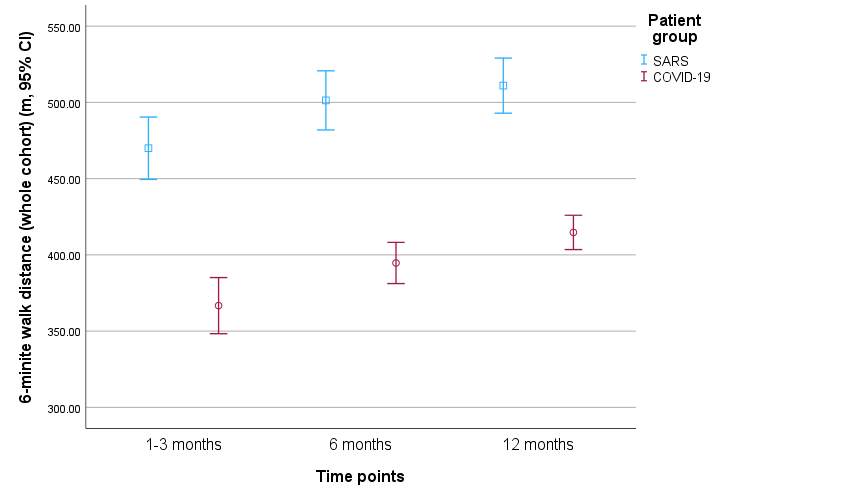


Within group difference:
9.7 (-4.4 to 23.8), 0.175

Within group difference:
31.4 (10.6 to 52.2), 0.004

Between-group difference:
from 1-3 to 6 months: -13.6 (-38.6 to 11.5), 0.286
from 6 to 12 months: 8.1 (-9.2 to 25.5), 0.356

Within group difference:
17.8 (7.5 to 28.1), 0.001

Within group difference:
17.8 (3.5 to 32.1), 0.015

Figure 2C. 6-minute walking distance of the whole cohort


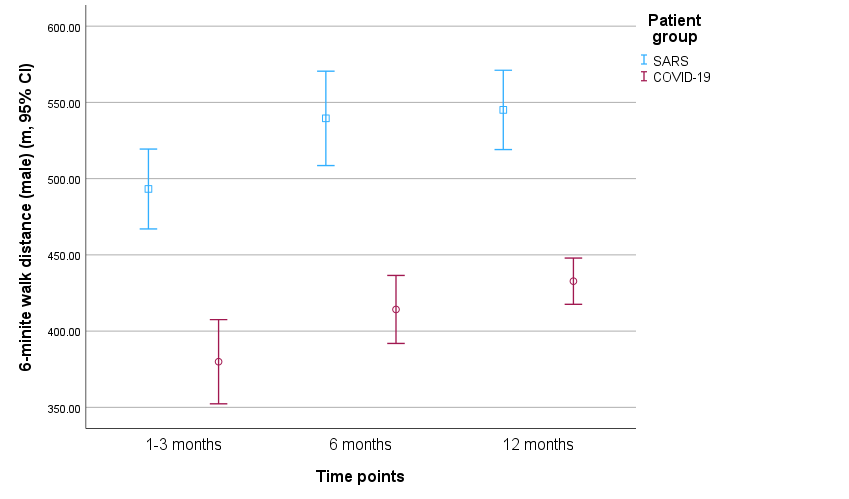


Within group difference:
46.3 (22.1 to 70.6), <0.001

Between-group difference:
from 1-3 to 6 months: -30.9 (-65.2 to 3.4), 0.077
from 6 to 12 months: 6.8 (-15.0 to 28.3), 0.542

Within group difference:
12.2 (-1.8 to 26.3), 0.086

Within group difference:
15.4 (-7.6 to 38.5), 0.180

Within group difference:
5.5 (-11.7 to 22.8), 0.519

Figure 2D. 6-minute walking distance of male survivors


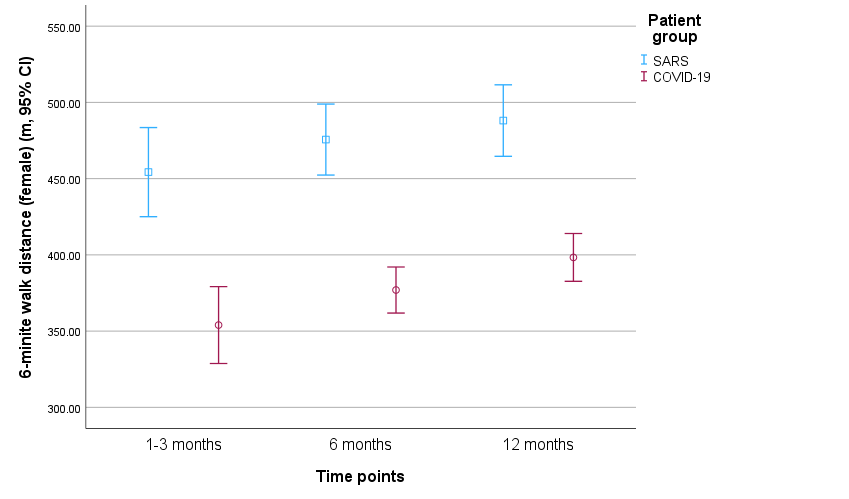


Within group difference:
12.5 (-8.4 to 33.3), 0.237

Within group difference:
22.8 (7.6 to 38.0), 0.004

Between-group difference:
from 1-3 to 6 months: -1.2 (-36.8 to 34.5), 0.948
from 6 to 12 months: 10.3 (-15.2 to 35.9), 0.425

Within group difference:
20.2 (1.8 to 38.6), 0.033

Within group difference:
21.3 (-9.8 to 52.4), 0.175

Figure 2E. 6-minute walking distance of female survivors


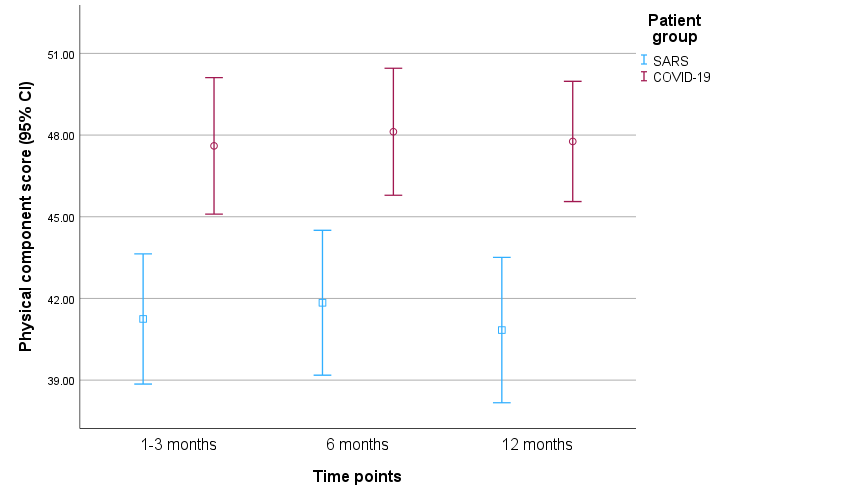


Within group difference:
-0.4 (-2.0 to 1.3), 0.666

Between-group difference:
from 1-3 to 6 months:
-0.2 (-2.9 to 2.4), 0.866
from 6 to 12 months:
0.6 (-1.6 to 2.9), 0.576

Within group difference:
-1.0 (-2.6 to 0.6), 0.213

Within group difference:
0.6 (-1.5 to 2.7), 0.578

Within group difference:
0.4 (-1.3 to 2.0), 0.656

Figure 2F. Physical component score


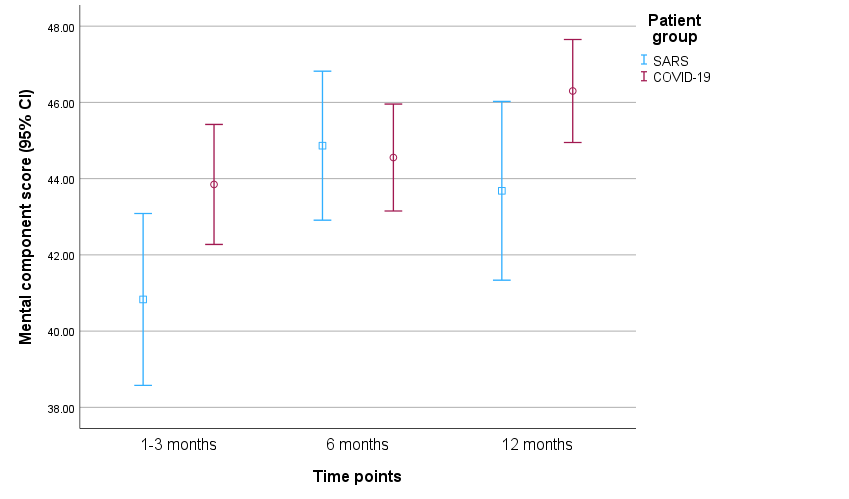


Between-group difference:
from 1-3 to 6 months:
-3.1 (-5.5 to -0.7), 0.012
from 6 to 12 months:
2.9 (0.8 to 5.1), 0.007

Within group difference
(COVID-19):
0.9 (-0.6 to 2.4), 0.219

Within group difference
(COVID-19):
1.7 (0.6 to 2.9), 0.003

Within group difference
(SARS):
-1.2 (-3.0 to 0.6), 0.198

Within group difference
(SARS):
4.0 (2.1 to 6.0), <0.001

Figure 2G. Mental component score

Figure 2. Within-group and between-group differences in serial changes of various physiological parameters and health-related quality of life between COVID-19 and SARS survivors at different time points. Differences are shown in mean ± standard deviation (95% confidence interval) followed by *p*-value. A: FEV_1_, B: FVC; C: 6-minute walking distance of the whole cohort; D: 6-minute walking distance of male survivors; E: 6-minute walking distance of female survivors; F: physical component score; G: mental component score.

*CI: confidence interval; COVID-19: coronavirus disease 2019; FEV_1_: forced expiratory volume in 1 second; FVC: forced vital capacity; SARS: severe acute respiratory syndrome*
